# The Predictive Validity of the Strange Situation Procedure: Evidence from Registered Analyses of Two Landmark Longitudinal Studies

**DOI:** 10.1017/S0954579423001487

**Published:** 2023-12-13

**Authors:** Marissa Nivison, Paul D. Caldo, Sophia W. Magro, K. Lee Raby, Ashley M. Groh, Deborah Lowe Vandell, Cathryn Booth-LaForce, R. Chris Fraley, Elizabeth A. Carlson, Jeffry A. Simpson, Glenn I. Roisman

**Affiliations:** 1 Institute of Child Development, University of Minnesota, Minneapolis, MN, USA; 2 Department of Psychology, University of Utah, Salt Lake City, UT, USA; 3 University of Missouri, Columbia, MO, USA; 4 School of Education, University of California Irvine, Irvine, CA, USA; 5 University of Washington, Seattle, WA, USA; 6 University of Illinois Urbana-Champaign, Urbana, IL, USA; 7 Department of Psychology, University of Minnesota, Minneapolis, MN, USA

**Keywords:** Infant attachment, strange situation procedure, academic skills, socioemotional outcomes

## Abstract

Meta-analyses demonstrate that the quality of early attachment is modestly associated with peer social competence (*r* = .19) and externalizing behavior (*r* = −.15), but weakly associated with internalizing symptoms (*r* = −.07) across early development (Groh et al., *Child Development Perspectives, 11*(1), 70–76, 2017). Nonetheless, these reviews suffer from limitations that undermine confidence in reported estimates, including evidence for publication bias and the lack of comprehensive assessments of outcome measures from longitudinal studies in the literature. Moreover, theoretical claims regarding the specificity of the predictive significance of early attachment variation for socioemotional versus academic outcomes had not been evaluated when the analyses for this report were registered (but see Dagan et al., *Child Development*, 1–20, 2023; Deneault et al., *Developmental Review, 70*, 101093, 2023). To address these limitations, we conducted a set of registered analyses to evaluate the predictive validity of infant attachment in two landmark studies of the Strange Situation: the Minnesota Longitudinal Study of Risk and Adaptation (MLSRA) and the NICHD Study of Early Child Care and Youth Development (SECCYD). Across-time composite assessments reflecting teacher report, mother report, and self-reports of each outcome measure were created. Bivariate associations between infant attachment security and socioemotional outcomes in the MLSRA were comparable to, or slightly weaker than, those reported in the recent meta-analyses, whereas those in the SECCYD were weaker for these outcomes. Controlling for four demographic covariates, partial correlation coefficients between infant attachment and all socioemotional outcomes were *r* ≤ .10 to .15 in both samples. Compositing Strange Situations at ages 12 and 18 months did not substantively alter the predictive validity of the measure in the MLSRA, though a composite measure of three different early attachment measures in the SECCYD did increase predictive validity coefficients. Associations between infant attachment security and academic skills were unexpectedly comparable to (SECCYD) or larger than (MLSRA) those observed with respect to socioemotional outcomes.

Central to attachment theory is the claim that children’s early experiences with caregivers become internalized as internal working models of the self, others, and the nature of relationships, which in turn influence developmental adaptation (Bowlby, 1969/1982). Building on the logic of Bowlby’s attachment theory, Ainsworth and colleagues (1978) developed the Strange Situation Procedure (SSP) – a key methodological innovation that made it possible to investigate the developmental sequelae of early attachment patterns. Through a series of separation and reunion episodes designed to activate the infant’s attachment behavioral system, Ainsworth established that the majority of infants in her original sample used their attachment figure(s) as a secure base from which to explore the environment when their caregiver was present and as a safe haven to effectively relieve their distress when reunited with a primary caregiver. Infants displaying this behavioral pattern were characterized as having a *secure* attachment to their caregivers. Ainsworth and colleagues also identified variations on these behavioral themes, with a minority of infants either ignoring their caregivers upon reunion (anxious *avoidance*) or demonstrating behavioral ambivalence by simultaneously signaling the desire for proximity yet not being effectively soothed by their caregivers (anxious *resistance*). Subsequently, Main and colleagues (Main & Hesse, 1990; Main & Solomon, 1986, 1990) described a subset of infants who, in addition to displaying secure, avoidant, or resistant behavioral patterns in the SSP, also displayed brief, anomalous behaviors suggestive of a “break-down” or *disorganization* with respect to their attachment-related strategies.

Ainsworth’s seminal work spurred five decades of research focused on the (mal)adaptive consequences of early attachment patterns. This now large corpus of studies was recently summarized via a series of meta-analyses, comprising the most comprehensive set of quantitative reviews of this literature to date. This meta-analytic work indicates that early attachment security as evaluated via observational assessments is associated with greater social competence with peers (*r* = .19; Groh et al., 2014), fewer behavior problems (*r* = −.15; Fearon et al., 2010), and somewhat fewer internalizing symptoms (*r* = −.07; Groh et al., 2012; see also Madigan et al., 2013).

## Limitations of the current empirical literature

Although quantitative reviews by Groh, Madigan, and others have been informative for drawing conclusions about the significance of infant attachment for socioemotional development, they also suffer from significant limitations. One of the major problems in this literature is the lack of registered analyses. *Registration* refers to a process in which the hypotheses, methods, or analytic plans (or any combination thereof) are documented publicly before the study or analyses begin (Lindsay et al., 2016). Registration is valuable because, when researchers are free to analyze their data in unconstrained ways, it is almost always possible to do so in a manner that leads to findings that can be interpreted in a theoretically coherent way (e.g., Simmons et al., 2011). However, such “findings” may be false positives or impossible to replicate. As such, they have the potential to interfere with the broader aims of rigorously building a cumulative knowledge base (Roisman, 2021).

Does the lack of registration have the potential to pose problems in the empirical study of infant attachment? There are at least three reasons to be concerned. First, meta-analyses in the broader field of attachment (Verhage et al., 2016) and on the question of how infant attachment is related to socioemotional outcomes in particular (Groh et al., 2017) both show signs of publication bias. This indicates that the published literature may not provide an accurate summary of how infant attachment is related to socioemotional outcomes.

Second, there are substantial researcher “degrees of freedom” (see Simmons et al., 2011) in the attachment literature, both on the predictor and on outcome side of the equation. On the predictor side, a typical research team needs to make decisions about how to quantify infant attachment: Should categories or dimensions be used? Should disorganized attachment be treated as a separate category? Should a simple secure versus insecure distinction be used? Should assessments taken across two or more time points be aggregated or should a “proportion of times classified as secure” be used? Each of these decisions is typically made *after* the data are collected and are being analyzed rather than beforehand. On the outcome side, a typical longitudinal study contains measures of an outcome of interest that may be assessed several times over development. Nonetheless, most reports focus on a small subset of those outcomes, creating opportunities for the data analysis to drive the decision-making rather than *a priori* theoretical ideas (see Gelman & Loken, 2014).

Finally, the majority of studies included in the Groh et al. (2017) meta-analyses were underpowered to detect the reported meta-analytic associations (median *N* = 44, 51, and 56 and median power for one-tailed tests = 37, 30, and 15% for studies on peer competence, externalizing, and internalizing outcomes, respectively). Although power is typically viewed as being a barrier to detecting true effects, at the level of the literature itself, low power tends to lead to a disproportionate number of false positives (see Ioannidis, 2005). This occurs because, in the presence of publication bias, the number of true effects relative to the number of false positives shrinks. This can create a situation in which, under reasonable assumptions, the published effect sizes for “real effects” are higher than they should be and the false positive rate in the literature greatly exceeds 5% (i.e., nominal alpha).

It is important to note that traditional meta-analysis does not solve these problems. That is, if a meta-analysis is conducted on underpowered studies that are subject to researcher degrees of freedom combined with publication bias, the meta-analysis will simply provide a robust estimate of biased associations. One key innovation of the present research is that we registered the critical decisions in this work before conducting the analyses: How attachment would be operationalized, what the outcomes would be, and what kinds of analyses would be conducted. Moreover, by drawing upon two of the largest and most comprehensive longitudinal data sets to date, this research provides a statistically powerful way to address one of the most enduring questions in the field of attachment: How strongly are the Strange Situation Procedure classifications related to subsequent socioemotional functioning? Moreover, we report all our results, regardless of whether they were supported by attachment theory’s claims.

Beyond researcher degrees of freedom, there are other limitations endemic to most quantitative reviews. On the one hand, the meta-analytic work in this area may *overestimate* the true effect sizes in this domain. This is due to a number of methodological limitations that affect meta-analyses generally, including the aggregation of mostly underpowered studies and the “file drawer problem” that exists in behavioral science literature (Rosenthal, 1979). Focusing on the meta-analyses by Fearon, Groh, and colleagues more specifically (Fearon et al., 2010; Groh et al., 2012, 2014, 2017), overestimation of the effect sizes is a possibility because those reviews did not take into account potential demographic confounders as potential “third variable” explanations for the associations documented between early attachment and the socioemotional outcomes.

On the other hand, it is possible that the meta-analyses in this area *underestimate* the true effect sizes due to a different set of methodological limitations. More specifically, in the Fearon et al. (2010) and Groh et al., (2012, 2014, 2017) meta-analyses, only the earliest measurement of the outcome variable was extracted when a study being reviewed measured the outcome variable at multiple time points. Additionally, the vast majority of the studies reviewed only assessed infant attachment at a single time point. Because multiple observations in aggregate increase the validity of a measure, the limitation of using one assessment of the predictor variable and one assessment of the outcome variable could have led to observed effect sizes that underestimate the true effects.

Beyond questions regarding the precise estimate of associations, another limitation of the meta-analyses concerns the narrow focus on socioemotional outcomes. Specifically, a key aim of meta-analyses in this area was to test the relative significance of early attachment across developmental domains (Groh et al., 2017). However, because outcomes with which attachment variation is generally not expected to be associated (e.g., academic outcomes) are rarely juxtaposed against findings related to socioemotional outcomes, the expected specificity of the predictive significance of early attachment had not been rigorously evaluated when the analyses for the current analysis were registered. More specifically, in contrast to the domains of social and emotional development, the domain of academic achievement has sometimes been cast as test of the discriminant validity of early measures of attachment. Indeed, early on, Sroufe (1988) anticipated very weak or even nil associations with objective measures of academic performance, but stronger associations with assessments of cognitive competence, when he noted:

“Ultimately, I expect no correlation with IQ, and to the extent that modest relationships are found with cognitive tests in the early years this may be because of different degrees of comfort with the examiner. Thus, in a sense, such relationships are indirect or even spurious. For the most part I would expect the unfolding of cognitive *competence* (in contrast to performance) to be robust with respect to attachment security” (pp. 26–27).

Although scholars such as Sroufe have suggested that attachment is unlikely to be associated with academic outcomes, other attachment scholars have long supported the view that attachment may be one developmental pathway to academic outcomes. Caregivers who promote secure attachment are more likely to support exploration and provide assistance in times of distress – factors that may contribute to higher academic achievement (see De Ruiter & Van IJzendoorn, 1993; see also Jerome et al., 2009). Given these opposing viewpoints, it is critical that the extent to which early attachment quality is associated with academic outcomes is empirically examined.

## The present study

Building on recently published meta-analytic reports by Groh and colleagues (2017), we conducted a set of analyses focused on the predictive validity of the Strange Situation Procedure specifically, leveraging two ongoing, landmark longitudinal studies that have examined the developmental consequences of infant attachment: the Minnesota Longitudinal Study of Risk and Adaptation (MLSRA) (Sroufe et al., 2005) and the Study of Early Child Care and Youth Development (SECCYD) (NICHD ECCRN, 2005). The MLSRA and the SECCYD both offer uniquely informative contexts in which to investigate the predictive significance of infant attachment across development. More specifically, the SSP was conducted when target participants in these cohorts were in infancy, and social competence, externalizing behaviors, internalizing symptoms, and academic skills data were acquired from multiple informants on those same participants at various time points from childhood into adulthood. In the current analyses, we aggregated all available outcome data, by informant, to maximize the reliability and validity of our assessments of each outcome domain. We also composited assessments of early attachment, though our focus here was primarily on the predictive validity of the SSP, specifically.

This analysis had three primary aims: (1) to assess the extent to which infant attachment security, assessed via the SSP, is associated with social competence, externalizing behaviors, and internalizing symptoms across childhood and early adulthood in the MLSRA and SECCYD, (2) to examine the extent to which attachment security is associated with academic outcomes across childhood and early adulthood in both longitudinal cohorts, and (3) to determine whether attachment security more strongly predicts socioemotional outcomes than academic skills in both longitudinal cohorts.

We also investigated whether the pattern of results hold when controlling for demographic covariates in follow-up analyses including analyses to determine to what extent the bivariate associations between the SSP and each outcome are significantly accounted for by the set of demographic variables. Given recent meta-analytic work revealing that early attachment is associated with higher levels of social competence (Groh et al., 2014), fewer externalizing behaviors (Fearon et al., 2010), and fewer internalizing symptoms (Groh et al., 2012; Madigan et al., 2013), we assumed that attachment security, as assessed by the SSP, would be positively associated with social competence and negatively associated with externalizing behaviors and internalizing symptoms, with the magnitude of effects paralleling those observed in the meta-analyses by Groh and colleagues. Regarding infant attachment and subsequent academic skills, we anticipated that a positive association would be observed. However, we were not well positioned to predict the relative magnitude of this effect size.

Importantly, our study rationale, aims, hypotheses, and analytic plan were all described in a detailed registration document^1^ published on the Open Science Framework prior to any of our analyses being conducted. The registration outlines an entire program of research on the predictive validity of the SSP in the MLSRA and SECCYD samples, whereas the present paper focuses on Aims 1 and 2 and parts of Aim 3 of the registration. The remaining registration aims focus on the comparative predictive significance of early attachment security and maternal sensitivity (elements of Aim 3) as well as how associations between early attachment and later indicators of adjustment are structured over time (Aim 4). Nonetheless, the current analysis serves as an omnibus and reasonably comprehensive examination of the predictive validity of the SSP in both the MLSRA and SECCYD, which to date have been analyzed in a rather piecemeal fashion in the literature. Although registration is no panacea to address all the issues and biases that are potentially present in meta-analyses, registration nonetheless is important in terms of being clear about one’s analytic plan, especially when one has prior knowledge of the data. This is because registration of a formal analytic plan promotes transparency around how decisions were made and makes it clear when researchers deviate from the script.

## Prior knowledge of data

There have been numerous studies conducted by our research group using the MLSRA and SECCYD data sets. The most relevant to this registration are those with a focus on the predictive significance of early maternal sensitivity in relation to the same outcomes to be studied here (e.g., Fraley et al., 2013; Haltigan et al., 2013; Raby et al., 2015, 2019; Roisman & Fraley, 2012). Because the current registered set of analyses is building on prior work with these data sets, the sets of outcome variables to be used in this study will be nearly identical to those used in the studies cited above, with caveats noted above and below. Importantly, results from both the MLSRA and SECCYD were also previously featured in the Fearon et al. (2010) meta-analysis as well as the Groh et al. meta-analyses of the predictive significance of early attachment security (2014; 2017; with which two coauthors were involved). However, these meta-analyses were restricted in focus to examining the bivariate association between the Strange Situations from these samples and the measurement of each outcome that occurred with the shortest temporal lag after the measurement of infant attachment, defaulting to maternal reports of the outcome measure when available. Thus, the existing meta-analyses present relatively limited information on the predictive validity of the Strange Situation in the MLSRA and SECCYD.

Although we have not worked on any other prior analyses examining the predictive significance of infant attachment for these specific outcome domains in either the MLSRA or SECCYD (but see Haltigan & Roisman, 2015), both investigations are considered landmark studies of the predictive significance of infant attachment and have produced many relevant papers. None of these reports to our knowledge, however, has explicitly examined comparatively, and in an omnibus fashion, the predictive validity of the Strange Situation across these four outcome domains, nor has there been a systematic examination of whether the predictive validity of the Strange Situation in these samples is robust to demographic covariates. At the time of preregistration (and before analyses), none of the authors were aware of how strongly early attachment security was associated with the four outcome domains in these data sets except by way of the limited data on these associations reflected in the meta-analyses by Fearon and colleagues (2010) and Groh and colleagues (2012, 2014, 2017). Authors were aware of how stable the dependent measures are within construct (by informant) from prior published analyses of the predictive significance of early maternal sensitivity (e.g., Raby et al., 2015). Despite prior meta-analytic work investigating the predictive significance of the Strange Situation Procedure, no single report to date has comprehensively examined the predictive validity of early attachment operationalized with multiple methods at multiple time points to socioemotional and academic outcomes through adulthood in the SECCYD and MLSRA samples.

## Method

### Participants

#### Minnesota Longitudinal Study of Risk and Adaptation (MLSRA)

The MLSRA (Sroufe et al., 2005) is an ongoing, landmark longitudinal study of development from infancy to adulthood. From 1975 to 1977, 267 pregnant mothers seeking free prenatal services through the local health department in Minneapolis, Minnesota were recruited. At the time of childbirth, 48% of the mothers of the target participants were teenagers, 65% were single, and 42% had completed less than a high school education. Approximately 79% of the mothers were White/non-Hispanic, 15% were African American, and 7% were Native American, Hispanic, or Asian American. Sixty-five percent of the children (i.e., target participants) were White/non-Hispanic, 17% were multiracial, 14% were African American, and 4% were Native American, Hispanic, or Asian American. The analytic sample for this study included children for whom (1) the SSP was completed at age 12 and/or 18 months, and (2) any social competence, externalizing behaviors, internalizing symptoms, or academic skills data were collected between ages 64 months and 39 years (*n* = 220). The follow-up of MLSRA and related analyses were approved by the University of Minnesota ethics review board (title: Early Life Stress, Developmental Processes, and Adult Health, IRB ID 1104S98312).

#### The NICHD Study of Early Child Care and Youth Development (SECCYD)

The SECCYD is another ongoing, landmark longitudinal study of human development. Full-term infants (*N* = 1,364) and their families were recruited in 1991 from hospitals based near 10 sites across major regions of the United States. Details about recruitment and selection procedures are available in prior publications from the study (see NICHD ECCRN, 2005). Two subsamples were the focus of this analysis of the SECCYD. The first subsample included participants for whom (1) the SSP was completed at 15 months, and (2) any social competence, externalizing behaviors, internalizing symptoms, or academic skills data were collected between ages 54 months and 18 years (*n* = 1,191; Booth-LaForce & Roisman, 2014; Vandell et al., 2016). This sample was used for our primary analyses. In supplemental analyses, a second analytic subsample was defined that included the children for whom (1) two out of the three available measures of early attachment (i.e., SSP at 15 months, Attachment Q-sort at 24 months, Modified SSP at 36 months) were completed, and (2) any social competence, externalizing behaviors, internalizing symptoms, or academic skills data were collected between 54 months and 18 years/end of high school (*n* = 1,196; Booth-LaForce & Roisman, 2014). Follow-up analyses included self-reported educational attainment from the 26-year assessment of the SECCYD (see Wegemer & Vandell, 2020). Although additional outcome data at around age 30 years recently became available in the SECCYD, these data were not analyzed here because the registration predated their availability. The follow-up of the SECCYD and related analyses were approved by the University of Minnesota ethics review board (title: “Follow-up of the NICHD Study of Early Child Care and Youth Development”; IRB ID 1207S16927).

The NICHD SECCYD data set is publicly available through the age 15-year assessment of the cohort^2^. Subsequent assessments of the SECCYD and the MLSRA data set are not publicly available. Study materials are available by contacting the corresponding authors.

### Early attachment measures

The early attachment variables used in this study were selected before data analyses began and were registered on June 12, 2020. Our primary focus in this report is the predictive significance of the classifications from the SSP. As such, in the MLSRA, the focal predictor variable was a composite of all available SSP data (at ages 12 and 18 months). In the SECCYD, the SSP was conducted only at 15 months; thus, infant attachment security at this single time point served as our focal predictor variable for this study.

In addition to our focal analyses, we also conducted follow-up (but registered) sensitivity analyses in the MLSRA that *disaggregated* the SSP composite into 12- and 18-month assessments. This was done to determine whether disaggregating the SSP data attenuates its predictive validity for socioemotional outcomes and academic skills. Given that the SECCYD, in contrast, contains measures of early attachment other than the SSP (i.e., the Attachment Q-Sort and the Modified SSP), we *aggregated* these measures to create a composite of early attachment (previously used by Groh et al., 2014; Booth-LaForce & Roisman, 2014) to determine whether compositing multiple early attachment measures strengthened their predictive validity in relation to socioemotional outcomes and academic skills. This composite served as our focal predictor variable in follow-up sensitivity analyses in the SECCYD.

#### Strange situation procedure

Infant-mother attachment quality was assessed in both the MLSRA and SECCYD using the SSP (Ainsworth et al., 1978). This laboratory procedure was designed to activate infants’ attachment system through a series of brief, moderately stressful episodes including caregiver-child separations and reunions. Infant-mother/caregiver attachment quality is based on the organization of the infant’s attachment behavior around the caregiver (mother), including the infant’s use of the caregiver (mother) as a secure base for exploration and a source of comfort following separation-related distress. Interactive relationship-based ratings of proximity seeking, contact maintenance, contact resistance, avoidance, and disorganization/disorientation serve as the basis for classification into one of four categories: Secure (B), Insecure-Avoidant (A), Insecure-Resistant (C), and Insecure Disorganized (D).

##### MLSRA.

In the MLSRA, 212 SSP assessments were conducted and available for coding at 12 months, whereas 197 assessments were conducted and available for coding at 18 months. Of these, a reduced number of the videos were available for subsequent disorganization/disorientation coding (*n* = 122 at 12 months; *n* = 83 at 18 months). Interrater agreement for 3-way classification (secure and anxious-avoidant and anxious-resistant) was 89% at 12 months and 92% agreement at 18 months. Interrater agreement for the disorganization classification across 12 and 18 months was 86%.

Consistent with classification guidelines used in prior meta-analyses, infants classified as avoidant, resistant, or disorganized were coded as insecure. If infants were primarily classified as disorganized but were also assigned a secondary classification of secure at a given assessment, they were coded as insecure for that assessment. Infants were classified separately for the 12- and 18-month assessments. In order to use all available data, a composite index indicating the *proportion of times secure* was created. A composite attachment variable was created to represent the percentage of times the infant-mother attachment relationship was classified as secure across the 12- and 18-month assessments (.00, .50, or 1.00). A small number of infant-mother dyads were classified secure at one assessment but did not participate at the other assessment (*n* = 16). Because infant attachment security was moderately stable from 12 to 18 months in the MLSRA, those cases were assigned a value on the composite variable of .75 to reflect the fact that we were not highly confident that these cases: (a) would have been classified as secure at the other assessment (1.00) or (b) would have been classified as insecure at the other assessment (.50). Likewise, cases that were classified as insecure at one assessment but were lacking data for the other assessment (*n* = 15) were assigned a value .25 on the composite variable.

Additionally, for the purpose of follow-up analyses, a dichotomous variable of Strange Situation security (1 = insecure, 2 = secure) was created by coding infants with B classifications as secure, and infants with A, C, and D classifications as insecure. This was done separately for the available 12- and 18-month Strange Situation data.

##### SECCYD.

In the SECCYD project, 1,191 infant-mother dyads completed the SSP at 15 months. Cases were classified using the four-way classifications. The SSP was administered by research assistants who had been trained in accordance with standard procedures (Ainsworth et al., 1978; Main & Solomon, 1990). A team of three trained and experienced research assistants coded the SSP assessments, and across all coder pairs, agreement with the four-way classification system was 83% (κ = .69). Per the preregistration and paralleling the approach taken in the MLSRA, a dichotomous variable of SSP security at 15 months was created that codes securely attached infants as secure and avoidant, resistant, and disorganized infants as insecure. Infants that were classified as “cannot classify” were also coded as insecure. If infants were classified as disorganized, but were assigned a secondary classification of secure, they were coded as insecure in these analyses. Based on this criterion, 60% of the infants were classified as secure (*n* = 710) and 40% were classified as insecure (*n* = 481). This dichotomous variable of security at 15 months served as the focal predictor variable in our analysis of the SECCYD.

#### Other measures of early attachment in the SECCYD

In addition to the SSP, SECCYD participants completed two other measures of early attachment: the Attachment Q-Set and the Modified SSP (more information about both of these measures is provided below). Given the variety of early attachment assessments collected in the SECCYD, a composite measure of early security, reflecting the *proportion of times* a child was coded as secure, was created and used as the predictor variable in our follow-up analyses of the SECCYD. If data were available on two or more early attachment assessments (SSP at 15 months, Attachment Q-Set at 24 months, Modified SSP at 36 months), a proportion of times secure score was assigned by calculating the number of times the child was classified securely attached for each available attachment assessment and dividing it by the total number of attachment assessments available for that child. This composite is identical to the one used by Groh and colleagues (2014).

##### Attachment Q-Set.

Child-mother attachment security was assessed at 24 months in 1,197 dyads using the Attachment Q-Set (E. Waters & Deane, 1985). Two-hour home observations were conducted. Afterwards, trained research assistants sorted the 90 items of the Q-sort into nine piles ranging from least to most characteristic of each participant. The sort summarized the child’s behavior as observed during the home visit, and this profile was correlated with the Security Criterion Sort to obtain a security score for each participant. A Pearson correlation of .30 or greater was rated as secure, which is reflective of the proportion of infants typically rated as secure when using the SSP, consistent with field recommendations (see Waters, 2003). Based on this, 47% of children were rated as insecure (*n* = 532) and 53% were rated as secure (*n* = 635). Across all research assistants, interrater reliability determined by ICC was .96.

##### Modified strange situation procedure.

Child-mother attachment security was assessed at 36 months in 1,140 dyads using the modified SSP developed by Cassidy and colleagues (1992). Cases were classified using the standard classifications of secure (B), insecure-avoidant (A), insecure-resistant (C), and disorganized (D). As was done with the traditional SSP, a dichotomous variable of security at 36 months was created, with infants with B classifications coded as secure and infants with A, C, and D classifications coded as insecure. Infants who were classified as “cannot classify” were also coded as insecure. If infants were classified as D, but were assigned a secondary classification of B, they were coded as insecure. Based on this, 62% of children were considered securely attached (*n* = 701) and 38% were coded insecure (*n* = 439). Intercoder agreement on the A, B, C, and D classification system was 75.7% (κ = .58).

### Outcome measures

The outcome measures and variables highlighted in this report were also predetermined in the registration (see Caldo et al., 2020). We selected variables that had been highlighted in prior work on the MLSRA and SECCYD cohorts that focused on the predictive significance of early maternal sensitivity (e.g., Fraley et al., 2013; Raby et al., 2015), allowing us to parallel the approach taken in prior work.

For socioemotional outcome measures, multiple informants were available in the form of mother-, teacher-, and self-reports. When possible, all data from multiple informants were leveraged in our analyses of both the MLSRA and SECCYD by compositing all available data separately by each informant. In the MLSRA, social competence data were only available from teachers, whereas symptoms of psychopathology data were available from all three informants. For the SECCYD, social competence and symptoms of psychopathology data were available from all three informants.

Academic skills outcomes in the MLSRA were limited to objective measures, whereas in the SECCYD, objective measures and teacher-reported measures were available and leveraged in our focal analyses. In both data sets, heterotypic indicators of academic skills were available in the form of self-reported educational achievement in early adulthood. Per the registration, these were utilized only in secondary, follow-up sensitivity analyses and, in that context, were included in composites of objective academic skills.

#### Social competence

##### MLSRA.

Teachers reported on participants’ social competence in the MLSRA by ranking each child’s competence with peers against their current classmates, according to how well each child in the classroom matched developmentally appropriate descriptions of social competence (see Sroufe et al., 1999, for more information). Teacher-reported social competence was collected during Kindergarten; Grades 1, 2, 3, and 6; and at age 16 years. These were the exact variables used to assess social competence in the MLSRA in prior publications focused on the predictive significance of early maternal sensitivity and abuse/neglect in the MLSRA (e.g., Raby et al., 2015, 2019). Available teacher-reported rankings were initially standardized by dividing participants’ ranks by the number of students in their class. These rankings were then averaged across all time points to create a composite that was used in our analyses (*α* = .78).

To assess social competence in a developmentally appropriate manner in young adulthood, competence within romantic relationships was used as a heterotypic marker of social competence. Romantic relationships were assessed using a semi-structured interview at ages 23 and 32 years which were averaged to create one composite measure of social competence in adulthood (*α* = .75). Interviews were evaluated by trained coders on the Relationship Effectiveness Scale, with higher scores reflecting more competent relationship engagement (see Englund et al., 2011). These were likewise the exact variables used to assess social competence during adulthood in prior MLSRA publications (e.g., Raby et al., 2015, 2019), though the romantic relationship data were not used in our primary analyses; instead, they were incorporated in sensitivity analyses, per the preregistration.

##### SECCYD.

Teachers, mothers, and target participants reported on participants’ social competence in the SECCYD using the Social Skills Questionnaire (SSQ) from the Social Skills Rating System (SSRS; Gresham & Elliott, 1990). Specifically, teachers completed the school version of the SSQ from the SSRS when children were in Kindergarten as well as Grades 1, 2, 3, 4, 5, and 6. To obtain an overall measure of social skills, items 1–30 which focused on behaviors such as cooperation, assertion, and self-control, from the SSQ were summed to create an overall measure of social competence. Higher scores indicated more socially skilled children (*α*s ranging from .93 to .94; *M* = .94). Similarly, mothers completed the 38-item SSQ from the SSRS when children were age 54 months; in Kindergarten; in Grades 1, 3, 4, 5, and 6; and ages 15 and 18 years. As with teacher reports, a measure of overall social skills was created by summing items 1–30 at each age to create an overall composite of social competence (*α*s ranged from .87 to .91, *M* = .89). These were the exact variables used to assess social competence in Fraley and colleagues (2013), apart from the addition of the mother reports of social skills at age 18 years, which became available after the 2013 paper was accepted for publication (though these data were featured in the related chapter by Roisman & Fraley, 2012).

In addition, participants self-reported on their own social competence using the SSQ from the SSRS at ages 15 and 18 years. Again, all items indexing social competence were summed to create a standardized scale of total social skills at each age (*α* = .91 at each assessment). All scores of total social skills were then averaged across all time points separately for teacher reports (*α* = .82), mother reports (*α* = .92), and self-reports (*α* = .67) to create composites that were used in our analyses.

#### Symptoms of psychopathology

##### MLSRA.

Teachers, mothers, and target participants reported on participants’ externalizing behaviors and internalizing symptoms. Teachers and mothers used the Teacher Report Form (TRF) and the Child Behavior Checklist (CBCL), respectively (Achenbach et al., 1987; Achenbach, 1991; Achenbach & Edelbrock, 1986). TRF assessments were collected at Kindergarten; Grades 1, 2, 3, and 6; and age 16 years. CBCL assessments were collected at age 64 months, Grade 1, and age 16 years. Additionally, participants self-reported on their externalizing behaviors and internalizing symptoms using the Youth Self-Report (YSR) at age 16 years, the Young Adult Self-Report (YASR) at ages 23 and 26 years, and the Adult Self-Report (ASR) at ages 32 and 39 years (Achenbach, 1997; Achenbach & Rescorla, 2001, 2003). These represent all mother, teacher, and self-reported Achenbach-based data available.

The standard externalizing behaviors scale (T scores) was used for this study. This scale includes items from each TRF, CBCL, and self-report (i.e., YSR, YASR, and ASR) assessment that tap aggressive and delinquent behavior. Externalizing behaviors showed adequate internal consistency across time for the TRF assessments (*α*s ranging from .93 to .96, *M* = .95), the CBCL assessments (*α*s ranging from .89 to .93 *M* = .91), and the self-reported assessments (*α*s ranging from .85 to .90, *M* = 0.88). T scores on externalizing behaviors were averaged across all available assessments by informant to create composites scores for teacher-reported TRFs (*α* = .81), mother reported CBCLs (*α* = .71), and participant self-reported YSR, YASRs, and ASRs (*α* = .85).

The standard internalizing scale (T scores) was also used. This scale includes items from each TRF, CBCL, and self-reported (i.e., YSR, YASR, and ASR) assessment that tap behaviors related to social withdrawal, anxiety, and depression. Internalizing problems had adequate internal consistency for teacher-reported TRF assessments (*α*s ranging from .82 to .90, *M* = .87), mother-reported CBCL assessments (*α*s ranging from .82 to .89, *M* = .86), and self-reported assessments (*α*s ranging from 0.88 to 0.94, *M* = 0.91). T scores on internalizing problems were averaged across all available assessments to create composites for teacher-reported TRFs (*α* = .65), mother-reported CBCLs (*α* = .73), and participant self-reported YSR, YASRs, and ASRs (*α* = .83).

##### SECCYD.

Teachers, mothers, and target participants reported on participants’ externalizing behaviors and internalizing symptoms. Teachers and mothers used the TRF and the CBCL, respectively (Achenbach et al., 1987; Achenbach, 1991; Achenbach & Edelbrock, 1986). TRF assessments were collected when participants were in Kindergarten as well as Grades 1, 2, 3, 4, 5, and 6. CBCL assessments were collected at 24, 36, and 54 months; Kindergarten; Grades 1, 3, 4, 5, and 6; and ages 15 and 18 years. Though the 24- and 36-month data were averaged in prior studies (e.g., Haltigan et al., 2013), we treated them as separate indicators in the current analyses given that we subsequently averaged all assessment points. Additionally, participants self-reported on their externalizing behaviors and internalizing symptoms using the YSR at ages 15 and 18 and at the end of high school assessments (Achenbach & Rescorla, 2001). The age 18 year and end of high school assessments overlapped partially, so those data were averaged to create a single assessment.

As with the MLSRA, the standard externalizing behaviors scale (T scores) was used for this study. The externalizing behaviors scale demonstrated adequate to high reliability across time for teachers’ TRFs (*α*s ranging from .94 to .95, *M* = .95), mothers’ CBCLs (*α*s ranging from .88 to .91, *M* = .89), and self-reported YSR, YASR, and ASR assessments (*α*s ranging from .86 to .88, *M* = .87). Externalizing scores were then averaged across all available assessments, separately for teacher-reported TRFs (*α* = .87), mother-reported CBCLs (*α* = .94), and participant self-reported YSR, YASRs, and ASRs (*α* = .73), to create composites used in our analyses.

Similarly, the standard internalizing scale (T scores) was also used. The internalizing problems scale demonstrated adequate reliability at each time point for teachers’ TRF assessments (*α*s ranging from .85 to .88, *M* = .86), mother’s CBCL assessments (*α*s ranging from .81 to .90, *M* = .84), and self-reported YSR, YASR, and ASR assessments (*α*s ranging from .89 to .92, *M* = .91). Just as we did with externalizing scores, internalizing scores from teacher-reported TRFs (*α* = .65), mother-reported CBCLs (*α* = .92), and participant self-reported YSR, YASRs, and ASRs (*α* = .70) were separately averaged across all available time points to create composites.

#### Academic skills

##### MLSRA.

Children’s objective academic skills during childhood and adolescence were assessed using the Peabody Individual Achievement Test (Dunn & Markwardt, 1970) during Grades 1, 2, 3, and 6. Subtest scores were highly correlated within each assessment (*α*s ranged from .86 to .91); thus, the total age-standardized score was used as an indicator of overall academic competence for each of these assessments. Later, participants completed the passage comprehension and calculation subtests of the Woodcock-Johnson Tests of Achievement (Woodcock & Johnson, 1989; Woodcock, 1990) at age 16 years. The standardized total scores (T scores) from the Peabody Individual Achievement Test and the standard scores of the passage comprehension and calculation subtests of the Woodcock-Johnson Tests of Achievement were *z*-standardized and then averaged across all time points to create a composite of objective academic skills (*α* = .92).

To assess the academic outcomes in a developmentally appropriate way in adulthood, participants’ self-reports of educational attainment at ages 23, 26, 28, 32, and 34 years were used as a heterotypic marker of academic skills. These were the exact markers used to assess academic skills in Raby and colleagues (2019). Additionally, self-reported educational attainment was recently assessed at ages 37 and 39 years. These reports were additionally included in this study. Participants’ self-reported educational attainment at each time point was coded on a 5-point scale, ranging from *no GED or high school diploma* to *4-year college degree or higher*. Consistent with our registration, the educational attainment data were not used in our primary analyses; however, these data were incorporated in follow-up analyses and web appendices. Educational attainment data was *z*-standardized within each time point, averaged across all time points, and included in a composite with the Peabody Individual Achievement Test and Woodcock-Johnson Tests of Achievement scores (*α* = .90).

##### SECCYD.

The Woodcock-Johnson Psycho-Educational Battery-Revised (WJ-R; Woodcock, 1990; Woodcock et al., 1989) was used as an objective measure of participants’ academic skills at 54 months; in Grades 1, 3, and 5; and at age 15 years. The standard scores for all available subscales of the WJ-R at each assessment point were averaged to create a measure of overall academic skills at each age (*α*s ranged from .81 to .91, *M* = .87). Additionally, teachers reported on participants’ academic skills using the SSRS (Gresham & Elliott, 1990) when children were in Kindergarten and at Grades 1, 2, 3, 4, and 5 (*α*s ranged from .95 to .96, *M* = .96). WJ-R standard scores were then averaged across all available time points to create an objective academic skills composite that was used in our main analyses (*α* = .94).

Additionally, a teacher-reported academic skills variable was created by summing items 31–39, which focused on academic success, from teacher reports using the SSRS at Kindergarten and Grades 1 through 6 (*α* = .91). Because this study builds on prior work from our research group on the SECCYD, the variables leveraged in this study are nearly identical to those featured in the study by Fraley and colleagues (2013) focused on the enduring versus transient predictive significance of early maternal sensitivity, with the exception that the more appropriate *standard scores* of the WJ-R were used instead of *W* scores in composites. Additionally, our use of these data differed in that teacher-reported academic skills and objective academic skills were (separately) averaged across all time points in our main analyses rather than studied by assessment.

To assess the academic domain in a developmentally appropriate way in adulthood, participants’ self-reported educational attainment at 26 years was used as a heterotypic marker of academic skills. Educational attainment data collected from participants at age 26 years was standardized and included in the objective academic skills composite that includes standardized WJ-R scores (*α* = .90). The subsequent composite including educational attainment was utilized only in follow-up analyses.

#### Covariates

##### MLSRA.

Variables that were previously used in analyses of the MLSRA (e.g., Raby et al., 2015) were used as covariates in our analyses: child sex, child race/ethnicity, maternal education, and socioeconomic status. Child sex was represented as a binary variable (1 = male, 2 = female). Child race/ethnicity was also represented as a binary variable (1 = White/non-Hispanic, 0 = other race and/or ethnicity) because most participants were White/non-Hispanic. Maternal education was represented as the number of years of education that the mother had completed. This was measured at two time points (3 months before birth and at 42 months) and was averaged to obtain a composite (*α* = .93). Socioeconomic status was assessed with Duncan’s Socioeconomic index (Stevens & Featherman, 1981), a broadly used measure of occupational standing and prestige, with scores based on primary caregivers’ occupational status at the 42-month assessment.

##### SECCYD.

Variables that are known to be associated with social competence, symptoms of psychopathology, and academic skills (see Fraley et al., 2013, Haltigan et al., 2013) were used as covariates in our SECCYD analyses: child sex, child race/ethnicity, maternal education, and socioeconomic status. This directly parallels the covariates used in our analyses of the MLSRA. Child sex was represented as a binary variable (1 = male, 2 = female). Child race/ethnicity was also represented as a binary variable (1 = White/non-Hispanic, 0 = other race and/or ethnicity) because most participants were White/non-Hispanic. Maternal education was represented as an ordered metric representing the number of years of education or the highest-level degree achieved. Socioeconomic status was operationalized as an income-to-needs ratio. This was collected at 6, 15, 24, and 36 months, and these individual variables were then averaged to create a mean income-to-needs index for early childhood (*α* = .94).

#### Power

Power calculations in multivariate models can be difficult to estimate because the power to detect any one parameter is dependent not only on the population value of the parameter in question, but all the parameters in the model at hand. Moreover, even in situations in which one can make reasonable assumptions about a parameter of interest, the other parameters require assumptions for which theory or data may not exist. Thus, for the sake of considering statistical power issues in the present work, we consider the power to detect simple bivariate associations – the building blocks of multivariate analyses. In the SECCYD, assuming a sample size of 1,191 and an alpha of .05, the statistical power to detect a population correlation of .10, .20, and .30 would be .93, >.99, and >.99, respectively. In the MLSRA, assuming a sample size of 197 and an alpha of .05, the statistical power to detect a population correlation of .10, .20, and .30 would be .29, .80, and >.99, respectively. Overall, these samples are well-powered to detect the kinds of associations that are commonly targeted in the developmental literature, with the exception of associations below a population *r* of .20 in the MLSRA.

## Results

Analyses are presented in three sections below, supported by extensive supplementary materials available online. Correlations among and descriptive data for all focal variables can be found in Table 1 for the MLSRA and in Table 2 for the SECCYD. As noted, analytic plans were registered and uploaded to the Open Science Framework (Caldo et al., 2020). Effect sizes were evaluated based on Funder and Ozer’s (2019) criteria (very small: *r* = .05–.09, small: *r* = .10–.19, medium: *r* = .20–.29, large: *r* = .30–.39, very large: *r* = .40 or greater).

### To what extent is infant attachment security, as assessed in the Strange Situation Procedure, associated with social competence, externalizing behaviors, and internalizing distress?

To determine the extent to which patterns of associations observed in the quantitative reviews summarized by Groh and colleagues (2017) could be reproduced in the MLSRA and SECCYD cohorts leveraging all available outcome data from those cohorts, zero-order correlations were estimated between infant attachment security and social competence, externalizing behaviors, and internalizing symptoms in each cohort (Aim 1). This was done separately by informant (i.e., mother-, teacher-, and self-report) as constrained by the availability of relevant data in each sample. Additionally, partial correlations were estimated to examine the associations between infant attachment security and all outcome variables, controlling for four demographic covariates (Aim 2). To further probe the role of demographic characteristics, we also conducted analyses to determine to what extent the bivariate associations between the SSP and each outcome were significantly accounted for by the set of demographic variables. In addition, we conducted Steiger’s (1980) Z comparisons across outcome domains, within informant (i.e., no cross-informant comparisons were conducted) to examine whether the bivariate and/or partial correlations differed in magnitude by outcome measure (Aim 2).

#### MLSRA

The focus of our primary analyses of the MLSRA data was on the proportion of times infants were observed to be secure with their maternal caregivers (based on all available SSP data; i.e., 12 and 18 months).

As reported in Table 1, bivariate associations between infant attachment and the socioemotional outcomes of interest were consistently comparable in magnitude to, though not notably larger than those reported in the Groh and colleagues meta-analyses (Fearon et al., 2010, Groh et al., 2012, 2014). More specifically, a medium-sized association was found between infant attachment and social competence rated by teachers (*r* = .20, *p* < .05), which is nearly identical to the effect size observed in prior meta-analytic work (*r* = .19; Groh et al., 2014). In contrast, correlations observed between infant security and externalizing symptoms were modest (teacher-reported, *r* = −.05, *p* = .49; mother-reported *r* = −.14, *p* = .06; self-reported *r* = −.14, *p* = .05). Correlations between infant security and internalizing symptoms were also modest (teacher-reported, *r* = −.16, *p* < .05; mother-reported *r* = −.15, *p* < .05; self-reported *r* = −.13, *p* = .09).

Second, following our registration plan, we explored the degree to which these associations might be attenuated by controlling for the four plausible “third variable” demographic confounds by conducting partial correlations. These results are presented in Table 3. Across all these analyses, the partial correlations computed were *r* ≤ ∼.14 and all non-significant.

Third, we conducted Steiger’s (1980) Z comparisons within informant. There was no evidence that the correlations and partial correlations differed by socioemotional outcome measures (these results are reported in detail in Table 5).

##### Follow-up analyses.

The analyses described above were then repeated with the proportion of times secure variable disaggregated into 12- and 18-month attachment security to assess the effects of compositing. The results of these secondary analyses were not materially different from what was observed with the proportion of times secure composite (see Table 1 for bivariate correlations; other analyses are available in Supplementary Tables 1–4). This pattern of results also held when using the heterotypic markers of social competence including adult relationship effectiveness (see Supplementary Tables 12 and 13).

Finally, we examined the extent to which the demographic covariates accounted for the association between infant attachment (as assessed with the proportion of times securely attached composite variable) and socioemotional outcomes. These results are presented in Supplementary Table 16. Overall, the ratio of the indirect to the total effect was 39% for teacher-reported social competence, 43–71% for externalizing behaviors, and 0–27% for internalizing symptoms. This general pattern of results held when the 12-month Strange Situation (Supplementary Table 17) and the 18-month Strange Situation (Supplementary Table 18) were treated as their own as predictors as well as when the heterotypic marker of social competence was examined (Supplementary Table 18).

#### SECCYD

Given our primary focus on the predictive validity of the SSP, the 15-month SSP was the focus of our main analyses in the SECCYD. As reported in detail in Table 2, bivariate associations in the SECCYD were weaker than those reported by Groh and colleagues (2017). More specifically, infant attachment security at age 15 months was weakly correlated with social competence (*rs* = .06–.09), externalizing behaviors (*r*s = −.01 to −.08), and internalizing problems (*rs* = −.01 to −.10) across informants. As was the case for the MLSRA, partial correlations that controlled for demographic covariates were even smaller in magnitude (see Table 4). Likewise, Steiger’s Z comparisons (presented in Table 6) did not reveal any consistent evidence that, within informant, bivariate or partial associations were stronger or weaker for any of the three socioemotional outcomes. This pattern of results held when analyses were re-run with the age 15-month SSP in which “cannot classify” cases were excluded from the analysis (see Supplementary Tables 14 and 15).

##### Follow-up analyses.

In follow-up analyses, presented in Supplementary Table 5–7, we used the early attachment composite variable reflecting the *proportion of times* a child was rated secure (as assessed with the SSP at age 15 months, the Attachment Q-sort at 24 months, and the Modified SSP at age 36 months). Bivariate associations are summarized in Supplemental Table 5. They indicate that the proportion of times secure variable revealed stronger associations between early attachment and socioemotional outcomes than did the 15-month SSP variable on its own. Specifically, medium-sized effects were found between early attachment and social competence rated by teachers and mothers (*r* = .23, *p* < .05 and *r* = .22, *p* < .05, respectively), with a very small effect for self-reported social competence (*r* = .09, *p* < .05). Small-to-medium associations were observed for teacher- and mother reports of externalizing behaviors (*r* = −.21, *p* < .05 and *r* = −.15, *p* < .05, respectively) and internalizing symptoms (*r* = −.21, *p* < .05 and *r* = −.13, *p* < .05, respectively). Self-reported externalizing and internalizing symptoms were not significantly correlated with proportion of times secure. Partial correlations (accounting for demographics covariates) for teacher- and mother-reported outcomes were smaller in magnitude but remained larger than the correlations and partial correlations associated with the 15-month SSP variable on its own (Supplementary Table 6). Self-reported externalizing and internalizing symptoms remained non-significant. Steiger’s Z comparisons, within informant, were not materially different from what was found with the 15-month SSP variable (Supplementary Table 7).

Finally, we examined the extent to which the demographic covariates accounted for the association between infant attachment as assessed with the SSP at 15 months and socioemotional outcomes. These results are presented in Supplementary Table 20. Overall, the ratio of the indirect to the total effect ranged from 29 to 60% for social competence, 13 to 60% for externalizing behaviors, and 0 to 100% for Internalizing symptoms. This overall pattern of results held when using the proportion of times secure variable (Supplementary Table 21) and when the “cannot classify” cases were dropped from the 15-month SSP (Supplementary Table 23).

### To what extent is infant attachment security, assessed in the Strange Situation Procedure, associated with academic skills?

Because no recent meta-analytic studies examining early attachment and academic skills had been conducted when this work was registered, another aim of this study was to better understand the extent to which attachment is associated with academic outcomes across development. To accomplish this, zero-order correlations were estimated between early attachment and all available measures of academic skills in the MLSRA and SECCYD cohorts. As with socioemotional outcomes, academic data were averaged across time and within informant.

#### MLSRA

As reported in Table 1, bivariate associations from our main analyses (the 12- and 18-month SSP composite) revealed a large, positive association between early attachment and objectively measured academic skills (*r* = .34, *p* < .05). This association remained statistically significant and was medium in terms of effect size after controlling for demographic covariates (*r* = .25, *p* < .01).

##### Follow-up analyses.

When 12- and 18-month SSP security were disaggregated in follow-up analyses, similar associations emerged (see Table 1 for bivariate associations and Supplementary Tables 1 and 2 for partial correlations). This pattern of results also held when using the heterotypic markers of academic skills that included adult self-reports of educational attainment (see Supplementary Tables 8). Finally, to further address Aim 2, we examined the extent to which the demographic covariates accounted for the association between infant attachment security and academic achievement. These results are presented in Supplementary Table 16. Overall, the ratio of the indirect to the total effect was 34%. This overall pattern of results held when the proportion of times secure variable was decomposed into the 12-month SSP (Supplementary Table 17) and the 18-month SSP (Supplementary Table 18), as well as when the heterotypic marker of academic achievement was examined (Supplementary Table 18).

#### SECCYD

Reported in Table 2 are bivariate associations from our main analysis of the SECCYD (with 15-month SSP as the predictor). The findings revealed very small, positive associations between early attachment and teacher-reported academic skills (*r* = .05, *p* = .11) and objectively measured academic skills (*r* = .07, *p* < .05). Partial correlations (accounting for demographics covariates) for both teacher-reported and objectively measured academic skills were even smaller in magnitude (*r*s = ≤ .03) and below the threshold for a very small effect based on Funder and Ozer’s (2019) criteria. This pattern of results held when analyses were re-run with the 15-month SSP in which “cannot classify” cases were excluded from analysis (see Supplementary Tables 14).

##### Follow-up analyses.

Follow-up analyses of the SECCYD, which leveraged the early attachment composite, produced notably larger positive associations than we found using the 15-month SSP on its own. Specifically, as reported in Supplementary Table 5, early attachment had a medium-sized, positive association with both teacher reports of academic skills (*r* = .21, *p* < .05) and objectively measured academic skills (*r* = .25, *p* < .05). Partial correlations for both teacher reports and objectively measured academic skills were weaker in magnitude, producing small effects (see Supplementary Table 6). In summary, as was the case with socioemotional outcomes in the SECCYD, compositing multiple measures of early attachment increased predictive validity.

Finally, we examined the extent to which the demographic covariates accounted for the association between infant attachment as assessed in the SSP at 15 months and academic achievement. These results are presented in Supplementary Table 20. Overall, the ratio of the indirect effect to the total effect was 71%. This pattern of results held when the “cannot classify” cases were dropped from the 15-month SSP (Supplementary Table 23) as well as when using the heterotypic marker of academic achievement (Supplementary Table 22). However, when using the proportion of times secure variable (Supplementary Table 21), the ratio of the indirect to the total effect was 48%.

### Does infant attachment security more strongly predict socioemotional outcomes than academic skills?

A final objective described in our registration was to examine how the magnitude of the associations between early attachment and socioemotional outcomes compare to those between early attachment and academic skills. As such, we conducted Steiger’s Z comparisons to examine whether early attachment is significantly more associated with socioemotional outcomes compared to academic skills in the MLSRA and SECCYD data sets, consistent with the claims of Sroufe (1988). Teacher reports and objective measures of academic skills were compared to socioemotional outcomes rated by all informants.

#### MLSRA

Findings from Steiger’s Z comparisons of the bivariate and partial correlations obtained from our main analyses of the MLSRA (with the proportion of times securely attached in 12- and 18-month SSPs as the predictor) are presented in Table 5. Taken together, these findings revealed that bivariate associations of infant attachment were larger for objective measures of academic skills than five of the seven socioemotional outcomes in the MLSRA. More specifically, objective academic skills were more strongly associated with infant attachment than: teacher-reported social competence (*Z* = −2.12, *p* < .05), externalizing behaviors (*Z* = −3.31, *p* < .05), and internalizing symptoms (*Z* = −2.44, *p* < .05), mother-reported externalizing behaviors (*Z* = −2.29, *p* < .05), and self-reported internalizing symptoms (*Z* = −2.09, *p* < .04). However, with the introduction of demographic covariates, infant attachment was no longer significantly more strongly associated with objective academic skills than with any socioemotional outcomes. When 12- and 18-month SSP security were disaggregated in follow-up analyses, Steiger’s Z comparisons revealed little evidence to suggest that academic outcomes were more strongly associated with infant attachment than were socioemotional outcomes, both with and without demographic covariates (see Supplementary Tables 3 and 4). Again, the pattern of results held with both the heterotypic markers of adult academic skills and adult social competence (see Supplemental Tables 9 and 13, respectively).

#### SECCYD

Table 6 reports detailed results of Steiger’s Z comparisons of the bivariate and partial correlations from our primary analyses of the SECCYD (which focused on the 15-month SSP as the predictor). Overall, we found little evidence that bivariate and partial correlations differed among the outcome variables. These results also held when examining the associations with the heterotypic marker of adult academic skills, as well as when SSP “cannot classify” cases were excluded from analysis (see Supplemental Tables 11 and 15, respectively).

In contrast, our supplemental analyses focused on the SECCYD early attachment composite revealed that early attachment was more strongly associated with both teacher-reported and objective academic skills compared to all three self-reported socioemotional outcomes, both with and without demographic covariates. Specifically, associations between early attachment security and both objective and teacher-reported academic skills were larger than those between early attachment security and self-reported social competence, externalizing behaviors, and internalizing symptoms (see Supplementary Table 7).

##### Preliminary parental sensitivity analyses.

The present analyses of the SSP were designed to examine the role of early attachment specifically in subsequent development. A relevant comparison would be to contrast such results with those based on direct observations of caregiving, using measures such as coded parental sensitivity, available on these same cohorts. As such, we conducted preliminary bivariate analyses that were a part of the larger program of research outlined in our preregistration, but not the focus of the present report, in order to examine the extent to which early caregiving experiences more generally predict socioemotional and academic outcomes. We operationalized observations of maternal sensitivity and socioemotional and academic outcomes in a manner consistent with work by Fraley and colleagues (2013) and Haltigan and colleagues (2013) in the SECCYD and by Raby and colleagues (2015) in the MLSRA. When we did so, associations between maternal sensitivity and socioemotional and academic outcomes were notably larger in magnitude than associations with the SSP. More specifically, in the MLSRA, early maternal sensitivity (assessed four times from 3 months to 42 months) was associated with teacher-reported social competence (*r* = .29), externalizing behaviors (teacher report, *r* = −.22; mother report, *r* = −.29, self-report, *r* = −.29), internalizing symptoms (teacher report, *r* = −.21; mother report, *r* = −.13, self-report, *r* = −.12), and academic achievement measured via the WJ-R and the PIAT (*r* = .47). In the SECCYD, early maternal sensitivity (assessed four times from 6 months to 36 months) was associated with social competence (teacher report, *r* = .29, mother report, *r* = .33, self-report, *r* = .17), externalizing behaviors (teacher report, *r* = −.34; mother report, *r* = −.24, self-report, *r* = −.10), internalizing symptoms (teacher report, *r* = −.21; mother report, *r* = −.18, self-report, *r* = −.01), and academic achievement measured via WJ-R (*r* = .47). These preliminary analyses were conducted to emphasize that early caregiving experiences, beyond the SSP, do demonstrate predictive significance for social and emotional adaptation across development.

## Discussion

Although meta-analytic work (Fearon et al., 2010; Groh et al., 2012, 2014) has been informative about the significance of infant attachment (in)security for socioemotional adjustment, this work is limited by the small sample sizes of studies included in these meta-analyses, evidence of publication bias in the literature, and researcher degrees of freedom. To overcome these issues, we conducted registered analyses of two landmark longitudinal studies of the legacy of early maternal attachments, here with a specific focus on the Strange Situation Procedure. More specifically, the current report builds directly on the programmatic meta-analytic research by Groh and colleagues (Fearon et al., 2010; Groh et al., 2012, 2014) to address three primary goals: (1) to better understand the extent to which early attachment security, as assessed via the SSP, is associated with social competence, externalizing behaviors, and internalizing symptoms; (2) to evaluate how strongly early attachment security, assessed via the SSP, is associated with academic skills; and (3) to evaluate whether or not early attachment security more strongly predicts socioemotional outcomes than academic skills.

In sum, in both the MLSRA and SECCYD, we found associations between infant attachment and socioemotional outcomes consistent with the correlations observed in meta-analyses focused on infant attachment and socioemotional development. Moreover, once accounting for demographic confounders, these associations were weaker than the effect sizes observed meta-analytically and modest in magnitude in the absolute sense (*r* ≤ ∼.10 to .15 in both studies when examining both bivariate associations and when controlling for demographic variables). To our surprise, security as assessed by the SSP was more strongly associated with academic skills than outcomes related to socioemotional development in the MLSRA. Moreover, a similar result was found in the SECCYD when we examined a composite assessment of early attachment in that data set.

### Links between infant attachment and socioemotional outcomes

One concern about the meta-analytic reviews of attachment security and socioemotional outcomes is that the bivariate associations estimated in that work may have *underestimated* the true strength of the associations between infant attachment and socioemotional outcomes. Findings from the current study are inconsistent with that possibility. First, none of the associations observed between infant attachment and socioemotional outcomes were markedly larger than those found in the quantitative reviews by Groh and colleagues (2012, 2014) and Fearon and colleagues (2010), despite our use of two landmark, rigorous studies of the consequences of early attachment. Second, once potential “third variable” confounders (i.e., demographic covariates) were accounted for, very small effects based on Funder and Ozer’s (2019) criteria (i.e., *r*s between .05 and .09) were generally observed between infant attachment and the socioemotional outcomes in both the MLSRA and SECCYD.^3^ Since multiple assessments of the socioemotional outcomes were composited in our study to increase measurement validity and reliability, these results suggest that the meta-analyses (Groh et al., 2012, 2014; Fearon et al., 2010) most likely *did not underestimate* the true strength of the associations because of less-than-ideal measurement of the outcome measures in the studies they quantitatively synthesized. It is important to note that although the MLSRA and the SECCYD were both included in prior meta-analyses, Groh and colleagues (2012, 2014) empirically examined whether the sample size of the SECCYD was driving the meta-analytic effects by conducting sensitivity analyses excluding the SECCYD. The authors found that the meta-analytic associations between early attachment and social competence and internalizing symptoms were not altered by the exclusion of the SECCYD. Therefore, the present study is not just a reiteration of the same data in the meta-analyses, and we can conclude that the findings from the present report are consistent with the meta-analytic associations between infant attachment and socioemotional outcomes even when the SECCYD was excluded from the meta-analytic analyses. Of course, it is the case that the meta-analyses only included the earliest time point of the outcome variables (i.e., socioemotional outcomes) and therefore the present analyses do contain more information from the MLSRA and SECCYD than was previously examined in the meta-analyses.

With that said, it would be a mistake to conclude from these results that early caregiving experiences in general have a relatively weak predictive significance for social and emotional adaptation across development. As we reported at the end of the Results section, preliminary analyses focused on maternal sensitivity in both the MLSRA and SECCYD data sets demonstrated reliably stronger associations with socioemotional outcomes than what was observed with respect to the SSP.

Although the SSP provides a snapshot of attachment-related experiences in the early life course, another approach is to collect multiple measures of attachment quality to better evaluate the quality of early attachment-relevant experiences. Our supplementary analyses of the SECCYD did reveal that compositing multiple measures of early attachment in this data set resulted in larger associations with subsequent social and emotional functioning. Specifically, we observed medium-sized effects between early attachment and social competence rated by teachers and mothers, and small-to-medium effects for teacher- and mother-reported externalizing behaviors and internalizing symptoms. After accounting for the effects of demographics and maternal sensitivity, teacher- and mother-reported outcomes were smaller in magnitude, but still remained larger than the correlations and partial correlations observed with only 15-month SSP attachment. These findings from the SECCYD data set provide further evidence that one way of potentially maximizing the predictive validity of measures of early attachment experiences is to assess early attachment multiple times across the first few years of life.

Unfortunately, at present, scholars in the field routinely use single assessments of attachment. This includes the vast majority of studies reviewed in prior meta-analyses examining the predictive significance of infant attachment. As the field translates attachment research in higher stakes contexts such as interventions (see Berlin et al., 2008) and within legal court proceedings (see Forslund et al., 2021), it will be important for researchers to aggregate multiple assessments over time and settings and improve the quality and standardized deployment of assessments of early attachment. Such practices will allow studies of early attachment to be more straightforwardly aggregated across time to maximize reliability and predictive validity (see Roisman & Groh, 2021).

### Infant attachment and the domain of academic competence

The domains of academic achievement and cognitive development have been seen historically as a test of the discriminant validity of measures of early attachment. Indeed, it has been suggested that attachment security should have very weak or even nil associations with objective measures of academic performance, but stronger associations with assessments of cognitive competence (Sroufe, 1988). In contrast to this view (but consistent with early dissenting voices; De Ruiter & Van IJzendoorn, 1993; Jerome et al., 2009) bivariate associations from the current study revealed a large association between infant attachment assessed in the SSP and objectively measured academic skills in the MLSRA. This association was robust, with a medium-sized effect still being observed after controlling for demographic covariates. Furthermore, bivariate associations between early attachment were consistently and significantly larger for objective measures of academic skills compared to socioemotional outcomes.

These findings are moreover consistent with two recent meta-analytic reports (not available at the time of our registration) that infant attachment is associated with academic outcomes (i.e., Dagan et al., 2023; Deneault et al., 2023). More specifically, Deneault and colleagues (2023) found that mother-child attachment, as measured by the Strange Situation Procedure, was meta-analytically associated with the child’s cognition (*r* = .17) and language outcomes (*r* = .16). Furthermore, in an individual participant data meta-analysis, children with two secure attachment relationships (i.e., to both mother and father) had higher language competence scores than those with only one or no secure attachment relationships (*d* = .26). Taken together, our findings – in combination with recent meta-analytic evidence – suggests that infant attachment is moderately associated with children’s later academic, cognitive, and language skills.

Importantly, a similar pattern has been observed repeatedly with respect to direct observations of the quality of early caregiving (e.g., maternal sensitivity), such that associations observed between the quality of mother-child interactions in infancy and academic skills is reliably stronger than it is for socioemotional outcomes across several prospective, longitudinal data sets (e.g., Fraley et al., 2013; Haltigan et al., 2013; Raby et al., 2015; Roisman & Fraley, 2012). Nevertheless, due to the relative lack of literature regarding infant attachment and academic skills (but see Dagan et al., 2023; Deneault et al., 2023), it is currently unclear what might be driving these patterns of results. Several hypotheses based on attachment theory have been proposed in earlier work, however (see Bretherton et al., 1979; Van IJzendoorn et al., 1995). First, the *attachment-teaching hypothesis* focuses on the secure parent-child dyad. Specifically, in a secure dyad, parents might be able to instruct their children and support the learning process more effectively than would be true of insecure parent-child dyads. Second, the *attachment-exploration hypothesis* emphasizes the active role of the child. Secure children may be more confident in using their primary caregiver as a secure base from which to explore exciting, as well as intimidating or threatening features of their environment, which could apply to academic environments as well. Third, the *social-network hypothesis* suggests that, because secure children tend to have more harmonious relationships with peers and less conflictual relationships with teachers (Magro et al., 2020; Sroufe et al., 2005), they may be able to use such relationships to garner and enhance cognitive stimulation both in and outside of the classroom. Fourth, the *attachment-cooperation hypothesis* suggests that secure children may be more likely to cooperate with testers of standardized tasks, perhaps resulting in better academic test performance. These proposed hypotheses are not mutually exclusive, and all of these models, along with the interplay between them (as well as the possibility of reverse causation), should be considered by researchers.

### Limitations and future directions

Although the present research leveraged data from two landmark longitudinal studies to investigate the significance of the SSP with respect to socioemotional and academic outcomes across development, this registered study is of course not without limitations. More specifically, despite both samples being moderate (MLSRA) and large (SECCYD) in size these cohorts were predominantly White and non-Hispanic. Future work would benefit from using more diverse and representative samples, both within and outside of the United States. Doing so would allow the field to learn more about how these processes work across different contexts. Although it is necessary to use data sets that collected attachment data 30+ years ago, if the goal is to study the long-term predictive effects of early attachment using prospectively gathered measures (e.g., the SECCYD and MLSRA cohorts were born in the mid-1970s and the early 1990s, respectively), future work should investigate more recent cohorts. Furthermore, a limitation of the MLSRA analysis is that not all videos were available to code for disorganization (as the SSP data were collected before the disorganization coding system was developed). Perhaps even more notably, the MLSRA and SECCYD focus only on *mother*-infant attachments. There is a clear need for additional studies that consider the role of father-child attachment or attachments to other primary or secondary caregivers in relation to both cognitive and emotional development, given the unique and interacting influences of each caregiver-child relationship (Dagan et al., 2021; Deneault et al., 2021; Magro et al., 2022).

In sum, the current research contributes to mounting evidence that the small-to-moderate associations between infant attachment and aspects of subsequent social and emotional (mal)adaptation reported meta-analytically by Groh and colleagues (2017) are consistent with the results of parallel analyses of adequately powered studies. Furthermore, the present results highlight the important predictive power of early attachment for subsequent academic performance. As noted by Roisman and Groh (2021), these results should prompt scholars to improve and, when possible, aggregate assessments of attachment relationships across childhood.

## Supplementary Material

1

## Figures and Tables

**Table 1.** Correlations, means, and standard deviations of infant attachment at 12 and 18 months, socioemotional outcomes (teacher-, mother-, and self-reports), and academic skills in the MLSRA

	1	2	3	4	5	6	7	8	9	10	11	12	13	14	15
1. Proportion of times secure	–														
2. Infant attachment, 12 months	**0.83**	–													
3. Infant attachment, 18 months	**0.82**	**0.34**	–												
4. Teacher-rated social competence	**0.20**	**0.20**	0.14	–											
5. Teacher-rated externalizing	−0.05	−0.02	−0.07	**−0.45**	–										
6. Teacher-rated internalizing	**−0.16**	−0.15	−0.13	**−0.62**	**0.41**	–									
7. Mother-rated externalizing	−0.14	−0.09	−0.14	**−0.34**	**0.39**	**0.24**	–								
8. Mother-rated internalizing	**−0.15**	−0.13	−0.11	**−0.25**	0.14	**0.28**	**0.69**	–							
9. Self-rated externalizing	−0.14	−0.11	−0.14	**−0.28**	**0.47**	**0.19**	**0.43**	**0.23**	–						
10. Self-rated internalizing	−0.13	−0.06	**−0.15**	**−0.18**	0.09	**0.23**	**0.20**	**0.26**	**0.61**	–					
11. Objective academic skills	**0.34**	**0.27**	**0.32**	**0.42**	**−0.23**	**−0.33**	**−0.17**	−0.10	0.01	0.01	–				
12. Child sex	0.07	0.07	0.05	**0.19**	−0.01	−0.07	0.05	0.08	−0.06	0.01	0.12	–			
13. Child race/ethnicity	0.07	0.02	0.10	0.09	**−0.26**	−0.09	0.05	0.02	−0.01	0.01	**0.20**	0.01	–		
14. Maternal education	**0.21**	**0.22**	0.13	**0.29**	**−0.21**	−0.14	**−0.31**	**−0.18**	**−0.26**	−0.02	**0.38**	−0.07	0.02	–	
15. Caregiver SEI	**0.17**	0.10	**0.18**	**0.16**	−0.11	−0.03	−0.08	−0.06	**−0.15**	−0.02	**0.28**	−0.06	0.13	**0.44**	–
*N*	220	212	197	190	189	189	188	188	184	184	190	220	220	219	185
*M*	0.53	1.52	1.55	49.97	55.13	53.46	57.44	54.76	53.04	50.45	−0.04	1.45	0.65	11.81	19.02
*SD* (%)	0.39	0.50	0.50	19.46	7.32	6.44	8.19	8.31	8.62	8.77	0.91	55%	65%	1.66	10.84

*Note.* Infant attachment was coded as 1 = secure, 0 = insecure. Child sex was coded as 1 = male, 2 = female. Race/ethnicity was coded as 1 = White/non-Hispanic, 0 = non-White or Hispanic. Bolded values indicate *p* < .05.

**Table 2.** Correlations, means, and standard deviations of infant attachment at 15 months, socioemotional outcomes (teacher-, mother-, and self-reports), and academic skills in the SECCYD

	1	2	3	4	5	6	7	8	9	10	11	12	13	14	15	16
1. Infant attachment, 15 months	–															
2. Teacher-rated social competence	0.06	–														
3. Teacher-rated externalizing	−0.05	**−0.68**	–													
4. Teacher-rated internalizing	**−0.10**	**−0.57**	**0.31**	–												
5. Mother-rated social competence	**0.09**	**0.42**	**−0.30**	**−0.25**	–											
6. Mother-rated externalizing	−0.01	**−0.36**	**0.43**	**0.17**	**−0.54**	–										
7. Mother-rated internalizing	−0.01	**−0.22**	**0.13**	**0.28**	**−0.43**	**0.70**	–									
8. Self-rated social competence	0.06	**0.26**	**−0.13**	**−0.16**	**0.28**	**−0.21**	**−0.13**	–								
9. Self-rated externalizing	**−0.08**	**−0.16**	**0.19**	**0.08**	**−0.15**	**0.28**	**0.14**	**−0.47**	–							
10. Self-rated internalizing	−0.04	**−0.06**	−0.01	**0.17**	**−0.08**	**0.12**	**0.21**	**−0.36**	**0.56**	–						
11. Objective academic skills	**0.07**	**0.45**	**−0.30**	**−0.25**	**0.34**	**−0.23**	**−0.13**	**0.22**	0.03	0.05	–					
12. Teacher-rated academic skills	0.05	**0.69**	**−0.42**	**−0.38**	**0.33**	**−0.25**	**−0.12**	**0.24**	**−0.07**	0.03	**0.73**	–				
13. Child sex	0.03	0.01	−0.03	0.01	−0.06	0.02	0.01	−0.01	0.02	0.05	0.03	−0.03	–			
14. Child race/ethnicity	−0.05	**−0.25**	**0.24**	**0.10**	**−0.23**	0.05	0.03	**−0.12**	**0.11**	0.03	**−0.31**	**−0.25**	0.00	–		
15. Maternal education	**0.07**	**0.33**	**−0.24**	**−0.21**	**0.28**	**−0.26**	**−0.17**	**0.16**	**−0.09**	−0.03	**0.49**	**0.38**	0.03	**−0.22**	–	
16. Income-to-needs ratio	0.05	**0.26**	**−0.18**	**−0.16**	**0.22**	**−0.19**	**−0.12**	**0.17**	**−0.12**	−0.04	**0.39**	**0.31**	0.04	**−0.22**	**0.53**	–
*N*	1191	1065	1065	1065	1095	1174	1174	918	941	941	1156	1064	1191	1191	1191	1189
*M*	0.60	103.05	50.58	49.58	104.37	48.84	48.61	110.41	50.56	48.26	104.54	98.87	1.49	0.22	14.38	3.52
*SD* (%)	0.49	10.45	7.03	5.71	12.51	7.80	7.27	13.97	9.17	9.39	11.18	9.95	52%	76%	2.48	2.69

*Note.* Infant attachment was coded as 1 = secure, 0 = insecure. Child sex was coded as 1 = male, 2 = female. Race/ethnicity was coded as 1 = White/non-Hispanic, 0 = non-White or Hispanic. Bolded values indicate *p* < .05.

**Table 3.** Partial correlations between proportion of times securely attached during infancy, socioemotional outcomes, and academic skills in the MLSRA

Predictor: Proportion of times securely attached	*r*	*p*
Teacher-reported outcomes		
Social competence	.10	.19
Externalizing	−.06	.47
Internalizing	−.07	.34
Mother-reported outcomes		
Externalizing	−.06	.28
Internalizing	−.14	.08
Self-reported outcomes		
Externalizing	−.08	.31
Internalizing	−.12	.11
Objectively measured outcomes		
Academic Skills	.25	<.01

*Note. n* = 170. Effects of demographics (i.e., child sex, child race/ethnicity, family socioeconomic status, and maternal education) partialed from associations.

**Table 4.** Partial correlations between infant attachment security assessed with the Strange Situation Procedure (SSP) at 15 months, socioemotional outcomes, and academic skills in the SECCYD

Predictor: 15-month SSP	*r*	*p*
Teacher-reported outcomes		
Social competence	.02	.60
Externalizing	−.04	.29
Internalizing	−.09	.01
Academic skills	.02	.63
Mother-reported outcomes		
Social competence	.06	.07
Externalizing	.01	.89
Internalizing	.01	.68
Self-reported outcomes		
Social competence	.06	.07
Externalizing	−.08	.02
Internalizing	−.04	.28
Objectively measured outcomes		
Academic skills	.03	.34

*Note. n* = 904. Effects of demographics (i.e., child sex, child race/ethnicity, family income-to-needs, and maternal education) partialed from associations.

**Table 5.** Steiger’s Z comparisons of associations between the proportion of times securely attached during infancy, socioemotional outcomes, and academic skills for the MLSRA

	Bivariate associations (*n* = 180)		Partial associations (*n* = 170)	
Comparisons	*Z*	*p*	*Z*	*p*
Teacher-reported outcomes				
Social competence – externalizing	1.81	.07	0.48	.63
Social competence – internalizing	0.82	.41	0.48	.63
Externalizing – internalizing	−1.10	.27	−0.12	.91
Social competence – objective academic skills	−2.12	.03	−1.77	.08
Externalizing – objective academic skills	−3.31	<.01	−1.86	.06
Internalizing – objective academic skills	−2.44	.02	−1.96	.05
Mother-reported outcomes				
Externalizing – internalizing	−0.51	.61	−1.36	.17
Externalizing – objective academic skills	−2.29	.02	−1.84	.07
Internalizing – objective academic skills	−1.86	.06	−1.06	.29
Self-reported outcomes				
Externalizing – internalizing	0.46	.64	−0.62	.53
Externalizing – objective academic skills	−1.80	.07	−1.71	.09
Internalizing – objective academic skills	−2.09	.04	−1.23	.22

*Note.* Bivariate associations = No covariates partialed from associations. Partial associations = Effects of demographics partialed from associations. Covariates include child sex, child race/ethnicity, socioeconomic status, and maternal education. Positive Z value indicates the left variable was larger. Negative Z value indicates the right variable was larger.

**Table 6.** Steiger’s Z comparisons of associations between infant attachment security assessed with the Strange Situation Procedure at 15 months, socioemotional outcomes, and academic skills for the SECCYD

	Bivariate associations (*n* = 905)		Partial associations (*n* = 904)	
Comparisons	*Z*	*p*	*Z*	*p*
Teacher-reported outcomes				
Social competence – objective academic skills	−0.58	.56	−0.26	.80
Social competence – teacher academic skills	0.00	1.00	0.00	1.00
Social competence – externalizing	−0.76	.44	−0.71	.48
Social competence – internalizing	−1.62	.10	−2.20	.03
Externalizing – internalizing	−0.77	.44	−1.23	.22
Externalizing – objective academic skills	0.00	1.00	0.24	.81
Externalizing – teacher academic skills	0.56	.58	0.52	.60
Internalizing – objective academic skills	0.74	.46	1.39	.16
Internalizing – teacher academic skills	1.36	.17	0.00	1.00
Mother-reported outcomes				
Social competence – objective academic skills	0.25	.80	0.69	.49
Social competence – teacher academic skills	0.77	.44	0.95	.34
Social competence – externalizing	2.20	.03	1.52	.13
Social competence – internalizing	1.69	.09	1.40	.16
Externalizing – internalizing	−0.39	.69	0.00	1.00
Externalizing – objective academic skills	−1.44	.15	−0.45	.65
Externalizing – teacher academic skills	−0.98	.33	−0.23	.82
Internalizing – objective academic skills	−1.13	.26	−0.43	.66
Internalizing – teacher academic skills	−0.68	.49	−0.22	.83
Self-reported outcomes				
Social competence – objective academic skills	0.24	.81	0.96	.34
Social competence – teacher academic skills	0.73	.46	0.93	.35
Social competence – externalizing	−0.59	.56	−0.57	.57
Social competence – internalizing	0.80	.42	0.53	.60
Externalizing – internalizing	1.61	.11	1.28	.19
Externalizing – objective academic skills	0.65	.52	1.29	.20
Externalizing – teacher academic skills	1.11	.27	1.30	.20
Internalizing – objective academic skills	−0.44	.66	0.22	.83
Internalizing – teacher academic skills	0.00	1.00	0.44	.66

*Note.* Bivariate associations = No covariates partialed from associations. Partial associations = Effects of demographics partialed from associations. Covariates include child sex, child race/ethnicity, family income-to-needs, and maternal education. Positive Z value indicates the left variable was larger. Negative Z value indicates the right variable was larger.

## Footnotes

1
https://osf.io/hu6g2/?view_only=a5fc263b462840eda48bcfcdaa82406e

2
https://www.icpsr.umich.edu/icpsrweb/ICPSR/studies/21940/summary

3 Of note, the analyses we present that control for covariates may be a less accurate assessment of the predictive validity of early attachment measures compared to the uncontrolled analyses insofar as those covariates – instead of being mere confounders – represent part of the developmental process that gives rise to the outcomes of interest (i.e., via setting the stage for secure versus insecure attachments, which in turn have downstream effects on developmental adaptation).
