# Supplementary material for: The Predictive Validity of the Strange Situation Procedure: Evidence from Registered Analyses of Two Landmark Longitudinal Studies
Source: Dev Psychopathol. Author manuscript; Available in PMC 2025 Aug 1. (PMC11169091; doi:10.1017/S0954579423001487)
Supplement: 1 [file NIHMS1946633-supplement-1.docx]

The Predictive Validity of the Strange Situation Procedure: Evidence from Registered Analyses of Two Landmark Longitudinal Studies

**SUPPLEMENTARY MATERIALS**

**Supplementary Table 1**

Bivariate and partial correlations between 12-month attachment security assessed with the Strange Situation Procedure, socioemotional outcomes, and academic skills in the MLSRA

|  | Bivariate associations  (*n* = 175) | | Partial associations  (*n* = 166) | |
| --- | --- | --- | --- | --- |
|  | *r* | *p* | *r* | *p* |
| Teacher-Reported Outcomes |  |  |  |  |
| Social Competence | .19 | .01 | .11 | .17 |
| Externalizing | .00 | .96 | -.09 | .25 |
| Internalizing | .11 | .17 | .05 | .51 |
|  |  |  |  |  |
| Mother-Reported Outcomes |  |  |  |  |
| Externalizing | .08 | .28 | .00 | .97 |
| Internalizing | .14 | .07 | .11 | .16 |
|  |  |  |  |  |
| Self-Reported Outcomes |  |  |  |  |
| Externalizing | .12 | .12 | .04 | .59 |
| Internalizing | .07 | .36 | .06 | .47 |
|  |  |  |  |  |
| Objectively Measured Outcomes |  |  |  |  |
| Academic Skills | .24 | < .01 | .17 | .03 |

*Note.* Bivariate associations = No covariates partialed from associations. Partial associations = Effects of demographics partialed from associations. Covariates include child sex, child race/ethnicity, caregiver socioeconomic status, and maternal education. Positive Z value indicates the left variable was larger. Negative Z value indicates the right variable was larger.

**Supplementary Table 2**

Bivariate and partial correlations between 18-month attachment security assessed with the Strange Situation Procedure, socioemotional outcomes, and academic skills in the MLSRA

|  | Bivariate associations  (*n* = 168) | | Partial associations  (*n* = 159) | |
| --- | --- | --- | --- | --- |
|  | *r* | *p* | *r* | *p* |
| Teacher-Reported Outcomes |  |  |  |  |
| Social Competence | .12 | .12 | .06 | .47 |
| Externalizing | -.06 | .41 | .00 | .98 |
| Internalizing | -.11 | .16 | -.06 | .45 |
|  |  |  |  |  |
| Mother-Reported Outcomes |  |  |  |  |
| Externalizing | -.14 | .07 | -.09 | .25 |
| Internalizing | -.13 | .10 | -.11 | .17 |
|  |  |  |  |  |
| Self-Reported Outcomes |  |  |  |  |
| Externalizing | -.15 | .06 | -.09 | .27 |
| Internalizing | -.15 | .06 | -.15 | .06 |
|  |  |  |  |  |
| Objectively Measured Outcomes |  |  |  |  |
| Academic Skills | .33 | < .01 | .26 | < .01 |
|  |  |  |  |  |

*Note.* Bivariate associations = No covariates partialed from associations. Partial associations = Effects of demographics partialed from associations. Covariates include child sex, child race/ethnicity, family socioeconomic status, and maternal education.

**Supplementary Table 3**

Steiger’s Z comparisons of associations between 12-month attachment security assessed with the Strange Situation Procedure, socioemotional outcomes, and academic skills in the expected direction for the MLSRA

|  | Bivariate associations  (*n* = 175) | | Partial associations  (*n* = 166) | |
| --- | --- | --- | --- | --- |
| Comparisons | *Z* | *p* | *Z* | *p* |
| Teacher-Reported Outcomes |  |  |  |  |
| Social Competence – Objective Academic Skills | -0.65 | .52 | -0.69 | .49 |
| Social Competence – Externalizing | 2.43 | .02 | 0.24 | .81 |
| Social Competence – Internalizing | 1.29 | .20 | 0.93 | .35 |
| Externalizing – Internalizing | -1.33 | .18 | 0.46 | .65 |
| Externalizing – Objective Academic Skills | -2.57 | .01 | -0.76 | .45 |
| Internalizing – Objective Academic Skills | -1.50 | .13 | -1.31 | .19 |
|  |  |  |  |  |
| Mother-Reported Outcomes |  |  |  |  |
| Externalizing – Internalizing | -1.02 | .31 | -1.82 | .07 |
| Externalizing – Objective Academic Skills | -1.66 | .10 | -1.58 | .11 |
| Internalizing – Objective Academic Skills | -1.00 | .32 | -0.56 | .57 |
|  |  |  |  |  |
| Self-Reported Outcomes |  |  |  |  |
| Externalizing – Internalizing | 0.76 | .45 | -0.31 | .76 |
| Externalizing – Objective Academic Skills | -1.16 | .25 | -1.28 | .20 |
| Internalizing – Objective Academic Skills | -1.62 | .11 | -1.01 | .31 |
|  |  |  |  |  |

*Note.* Bivariate associations = No covariates partialed from associations. Partial associations = Effects of demographics partialed from associations. Covariates include child sex, child race/ethnicity, family socioeconomic status, and maternal education. Positive Z value indicates the left variable was larger. Negative Z value indicates the right variable was larger.

**Supplementary Table 4**

Steiger’s Z comparisons of associations between 18-month attachment security assessed with the Strange Situation Procedure, socioemotional outcomes, and academic skills in the expected direction for the MLSRA

|  | Bivariate associations  (*n* = 168) | | Partial associations  (*n* = 159) | |
| --- | --- | --- | --- | --- |
| Comparisons | *Z* | *p* | *Z* | *p* |
| Teacher-Reported Outcomes |  |  |  |  |
| Social Competence – Objective Academic Skills | -2.71 | .01 | -2.28 | .03 |
| Social Competence – Externalizing | 0.75 | .46 | 0.69 | .36 |
| Social Competence – Internalizing | 0.16 | .88 | 0.00 | 1.00 |
| Externalizing – Internalizing | -0.59 | .55 | -0.69 | .49 |
| Externalizing – Objective Academic Skills | -2.85 | < .01 | -2.44 | .01 |
| Internalizing – Objective Academic Skills | -2.48 | .01 | -2.11 | .04 |
|  |  |  |  |  |
| Mother-Reported Outcomes |  |  |  |  |
| Externalizing – Internalizing | 0.17 | .87 | -0.32 | .75 |
| Externalizing – Objective Academic Skills | -2.01 | .04 | -1.61 | .11 |
| Internalizing – Objective Academic Skills | -2.01 | .05 | -1.39 | .16 |
|  |  |  |  |  |
| Self-Reported Outcomes |  |  |  |  |
| Externalizing – Internalizing | 0.15 | .88 | -0.90 | .37 |
| Externalizing – Objective Academic Skills | -1.73 | .08 | -1.65 | .10 |
| Internalizing – Objective Academic Skills | -1.84 | .07 | -1.01 | .31 |
|  |  |  |  |  |

*Note.* Bivariate associations = No covariates partialed from associations. Partial associations = Effects of demographics partialed from associations. Covariates include child sex, child race/ethnicity, family socioeconomic status, and maternal education. Positive Z value indicates the left variable was larger. Negative Z value indicates the right variable was larger.

**Supplementary Table 5**

Correlations, Means, and Standard Deviations of Proportion of Times Secure Ratings, Socioemotional Outcomes (Teacher-, Mother-, and Self-Reports), and Academic Skills in the SECCYD

|  | 1 | 2 | 3 | 4 | 5 | 6 | 7 | 8 | 9 | 10 | 11 | 12 | 13 | 14 | 15 | 16 |
| --- | --- | --- | --- | --- | --- | --- | --- | --- | --- | --- | --- | --- | --- | --- | --- | --- |
| 1. Proportion of Times Secure | — |  |  |  |  |  |  |  |  |  |  |  |  |  |  |  |
| 2. Teacher-Rated Social Competence | **0.23** | — |  |  |  |  |  |  |  |  |  |  |  |  |  |  |
| 3. Teacher-Rated Externalizing | **-0.21** | **-0.68** | — |  |  |  |  |  |  |  |  |  |  |  |  |  |
| 4. Teacher-Rated Internalizing | **-0.21** | **-0.56** | **0.31** | — |  |  |  |  |  |  |  |  |  |  |  |  |
| 5. Mother-Rated Social Competence | **0.22** | **0.42** | **-0.31** | **-0.24** | — |  |  |  |  |  |  |  |  |  |  |  |
| 6. Mother-Rated Externalizing | **-0.15** | **-0.36** | **0.43** | **0.17** | **-0.54** | — |  |  |  |  |  |  |  |  |  |  |
| 7. Mother-Rated Internalizing | **-0.13** | **-0.22** | **0.13** | **0.28** | **-0.43** | **0.71** | — |  |  |  |  |  |  |  |  |  |
| 8. Self-Rated Social Competence | **0.09** | **0.28** | **-0.14** | **-0.17** | **0.28** | **-0.21** | **-0.14** | — |  |  |  |  |  |  |  |  |
| 9. Self-Rated Externalizing | -0.05 | **-0.16** | **0.19** | **0.09** | **-0.16** | **0.28** | **0.14** | **-0.47** | — |  |  |  |  |  |  |  |
| 10. Self-Rated Internalizing | -0.01 | **-0.08** | -0.01 | **0.18** | **-0.08** | **0.13** | **0.21** | **-0.37** | **0.57** | — |  |  |  |  |  |  |
| 11. Objective Academic Skills | **0.25** | **0.45** | **-0.30** | **-0.25** | **0.34** | **-0.23** | **-0.13** | **0.22** | 0.03 | 0.05 | — |  |  |  |  |  |
| 12. Teacher-Academic Skills | **0.21** | **0.69** | **-0.42** | **-0.37** | **0.33** | **-0.25** | **-0.12** | **0.25** | **-0.08** | 0.02 | **0.73** | — |  |  |  |  |
| 13. Child Sex | 0.04 | 0.02 | -0.04 | 0.01 | -0.06 | 0.02 | 0.02 | 0.00 | 0.01 | 0.04 | 0.03 | -0.02 | — |  |  |  |
| 14. Child Race/Ethnicity | **-0.12** | **-0.25** | **0.24** | **0.10** | **-0.23** | 0.05 | 0.04 | -0.12 | **0.11** | 0.03 | **-0.32** | **-0.25** | 0.01 | — |  |  |
| 15. Maternal Education | **0.20** | **0.34** | **-0.24** | **-0.21** | **0.30** | **-0.27** | **-0.18** | **0.17** | **-0.10** | -0.04 | **0.49** | **0.39** | 0.03 | **-0.21** | — |  |
| 16. Income-to-needs Ratio | **0.14** | **0.26** | **-0.18** | **-0.16** | **0.23** | **-0.20** | **-0.14** | **0.16** | **-0.12** | -0.05 | **0.39** | **0.31** | 0.04 | **-0.22** | **0.53** | — |
| *N* | 1196 | 1080 | 1080 | 1080 | 1110 | 1194 | 1194 | 926 | 949 | 949 | 1156 | 1079 | 1196 | 1196 | 1196 | 1194 |
| *M* | 0.58 | 103.03 | 50.63 | 49.57 | 104.45 | 48.88 | 48.60 | 110.38 | 50.56 | 48.26 | 104.54 | 98.75 | 1.49 | 0.22 | 14.38 | 3.50 |
| *SD* | 0.31 | 10.42 | 7.01 | 5.72 | 12.58 | 7.82 | 7.29 | 13.99 | 9.21 | 9.40 | 11.18 | 9.95 | 0.50 | 0.41 | 2.48 | 2.67 |

*Note.* Child sex was coded as 1 = male, 2 = female. Race/ethnicity was coded as 1 = White/non-Hispanic, 0 = non-White or Hispanic. Bolded values indicate *p* < .05.

**Supplementary Table 6**

Bivariate and partial correlations between proportion of times securely attached, socioemotional outcomes, and academic skills in the SECCYD

|  | Bivariate associations  (*n* = 913) | | Partial associations  (*n* = 912) | |
| --- | --- | --- | --- | --- |
| Comparisons | *r* | *p* | *r* | *p* |
| Teacher-Reported Outcomes |  |  |  |  |
| Social Competence | .24 | < .01 | .18 | < .01 |
| Externalizing | -.24 | < .01 | -.18 | < .01 |
| Internalizing | -.21 | < .01 | -.17 | < .01 |
| Academic Skills | .21 | < .01 | .15 | < .01 |
|  |  |  |  |  |
| Mother-Reported Outcomes |  |  |  |  |
| Social Competence | .20 | < .01 | .15 | < .01 |
| Externalizing | -.13 | < .01 | -.09 | .01 |
| Internalizing | -.09 | < .01 | -.07 | .05 |
|  |  |  |  |  |
| Self-Reported Outcomes |  |  |  |  |
| Social Competence | .09 | .01 | .05 | .11 |
| Externalizing | -.06 | .05 | -.04 | .27 |
| Internalizing | -.01 | .71 | .00 | .93 |
|  |  |  |  |  |
| Objectively Measured Outcomes |  |  |  |  |
| Academic Skills | .24 | < .01 | .16 | < .01 |
|  |  |  |  |  |

*Note.* Bivariate Associations = No covariates partialed from associations. Partial Associations = Effects of demographics partialed from associations. Covariates include child sex, child race/ethnicity, family income-to-needs, and maternal education.

**Supplementary Table 7**

Steiger’s Z comparisons of associations between proportion of times secure ratings, socioemotional outcomes, and academic skills for the SECCYD

| Proportion of times Secure Associations | Bivariate associations  (*n* = 913) | | Partial associations  (*n* = 912) | |
| --- | --- | --- | --- | --- |
| Comparisons | *Z* | *p* | *Z* | *p* |
| Teacher-Reported Outcomes |  |  |  |  |
| Social Competence – Objective Academic Skills | 0.00 | 1.00 | 0.53 | .60 |
| Social Competence – Teacher Academic Skills | 1.20 | .23 | 1.08 | .28 |
| Social Competence – Externalizing | 0.00 | 1.00 | 0.00 | 1.00 |
| Social Competence – Internalizing | 1.02 | .31 | 0.32 | .75 |
| Externalizing – Internalizing | 0.79 | .43 | 0.25 | .80 |
| Externalizing – Objective Academic Skills | 0.00 | 1.00 | 0.48 | .63 |
| Externalizing – Teacher Academic Skills | 0.88 | .38 | 0.80 | .42 |
| Internalizing – Objective Academic Skills | -0.77 | .44 | 0.24 | .81 |
| Internalizing – Teacher Academic Skills | 0.00 | 1.00 | 0.53 | .59 |
|  |  |  |  |  |
| Mother-Reported Outcomes |  |  |  |  |
| Social Competence – Objective Academic Skills | -1.05 | .30 | -0.24 | .81 |
| Social Competence – Teacher Academic Skills | -0.26 | .79 | 0.00 | 1.00 |
| Social Competence – Externalizing | 2.24 | .03 | 1.83 | .07 |
| Social Competence – Internalizing | 3.19 | < .01 | 2.26 | .02 |
| Externalizing – Internalizing | 1.60 | .11 | 0.78 | .43 |
| Externalizing – Objective Academic Skills | -2.75 | .01 | -1.60 | .10 |
| Externalizing – Teacher Academic Skills | -2.01 | .04 | -1.42 | .16 |
| Internalizing – Objective Academic Skills | -3.49 | < .01 | -1.98 | .05 |
| Internalizing – Teacher Academic Skills | -2.80 | < .01 | -1.80 | .08 |
|  |  |  |  |  |
| Self-Reported Outcomes |  |  |  |  |
| Social Competence – Objective Academic Skills | -3.71 | < .01 | -2.54 | .01 |
| Social Competence – Teacher Academic Skills | -3.01 | < .01 | -2.38 | .02 |
| Social Competence – Externalizing | 0.88 | .38 | 0.29 | .77 |
| Social Competence – Internalizing | 2.14 | .03 | 1.33 | .18 |
| Externalizing – Internalizing | 1.63 | .10 | 1.30 | .19 |
| Externalizing – Objective Academic Skills | -3.95 | < .01 | -2.74 | .01 |
| Externalizing – Teacher Academic Skills | -3.39 | < .01 | -2.39 | .02 |
| Internalizing – Objective Academic Skills | -5.16 | < .01 | -3.89 | < .01 |
| Internalizing – Teacher Academic Skills | -4.38 | < .01 | -3.65 | < .01 |
| *Note.* Bivariate associations = No covariates partialed from associations. Partial associations = Effects of demographics partialed from associations. Covariates include child sex, child race/ethnicity, family income-to-needs, and maternal education. Positive Z value indicates the left variable was larger. Negative Z value indicates the right variable was larger | | | | |

**Supplementary Table 8**

Correlations between all study variables and the adult academic achievement variable in the MLSRA

|  | Bivariate associations  (*n* =180) | Partial associations  (*n* = 170) |
| --- | --- | --- |
| Proportion of Times Secure | **.28** | **.22** |
| Infant Attachment, 12mo | **.23** | **.18** |
| Infant Attachment, 18mo | **.25** | **.18** |
| Teacher-Rated Social Competence | **.52** | **.41** |
| Teacher-Rated Externalizing | **-.26** | -.12 |
| Teacher-Rated Internalizing | **-.31** | **-.27** |
| Mother-Rated Externalizing | **-.26** | -.12 |
| Mother-Rated Internalizing | **-.14** | -.11 |
| Self-Rated Externalizing | **-.11** | .05 |
| Self-Rated Internalizing | -.06 | -.06 |

*Note.* The adult academic achievement variable contains the childhood objective measures of academic achievement used in the main analyses plus the *z*-standardized self-reports of educational attainment in adulthood from 23, 26, 28, 32, 34, 37, and 39 years. Covariates include child sex, child race/ethnicity, family socioeconomic status, and maternal education. Bolded values indicate *p* < .05.

**Supplementary Table 9**

Steiger’s Z comparisons of associations between the proportion of times securely attached, socioemotional outcomes, and adult academic skills for the MLSRA

|  | Bivariate associations  (*n* = 180) | | Partial associations  (*n* = 170) | |
| --- | --- | --- | --- | --- |
| Comparisons | *Z* | *p* | *Z* | *p* |
| Teacher-Reported Outcomes |  |  |  |  |
| Social Competence – Adult Academic Skills | -1.41 | .16 | -0.97 | .33 |
| Externalizing – Adult Academic Skills | -2.70 | .01 | -1.19 | .23 |
| Internalizing – Adult Academic Skills | -1.76 | .08 | -1.18 | .24 |
|  |  |  |  |  |
| Mother-Reported Outcomes |  |  |  |  |
| Externalizing – Adult Academic Skills | -1.70 | .09 | -1.20 | .23 |
| Internalizing – Adult Academic Skills | -1.27 | .21 | -0.39 | .70 |
|  |  |  |  |  |
| Self-Reported Outcomes |  |  |  |  |
| Externalizing – Adult Academic Skills | -1.24 | .21 | -0.95 | .34 |
| Internalizing – Adult Academic Skills | -1.51 | .13 | -0.58 | .56 |
|  |  |  |  |  |

*Note.* Bivariate associations = No covariates partialed from associations. Partial associations = Effects of demographics partialed from associations. Covariates include child sex, child race/ethnicity, family socioeconomic status, and maternal education. Positive Z value indicates the left variable was larger. Negative Z value indicates the right variable was larger. The adult academic achievement variable contains the childhood objective measures of academic achievement used in the main analyses plus the *z*-standardized self-reports of educational attainment in adulthood from 23, 26, 28, 32, 34, 37, and 39 years.

**Supplementary Table 10**

Correlations between all study variables and the adult academic achievement variable in the SECCYD

|  | Bivariate Associations  *n* = 905 | Partial Associations  *n* = 904 |
| --- | --- | --- |
| Infant Attachment, 15mo | **.07** | .05 |
| Teacher-Rated Social Competence | **.49** | **.35** |
| Teacher-Rated Externalizing | **-.34** | **-.21** |
| Teacher-Rated Internalizing | **-.26** | **-.17** |
| Mother-Rated Social Competence | **.32** | **.18** |
| Mother-Rated Externalizing | **-.25** | **-.15** |
| Mother-Rated Internalizing | **-.13** | -.06 |
| Self-Rated Social Competence | **.25** | **.16** |
| Self-Rated Externalizing | -.03 | .06 |
| Self-Rated Internalizing | .04 | .08 |
| Teacher-Academic Skills | **.76** | **.69** |

*Note.* The adult academic achievement variable contains the childhood objective measures of academic achievement used in the main analyses plus the *z*-standardized self-report of educational attainment in adulthood at 26 years of age. Covariates include child sex, child race/ethnicity, family income-to-needs, and maternal education. Bolded values indicate *p* < .05.

**Supplementary Table 11**

Steiger’s Z comparisons of associations between 15-month Strange Situation Procedure, socioemotional outcomes, and adult academic skills for the SECCYD

|  | Bivariate associations  (*n* = 905) | | Partial associations  (*n* = 904) | |
| --- | --- | --- | --- | --- |
| Comparisons | *Z* | *p* | *Z* | *p* |
| Teacher-Reported Outcomes |  |  |  |  |
| Social Competence – Adult Academic Skills | -0.89 | .37 | -0.79 | .43 |
| Externalizing – Adult Academic Skills | -0.26 | .79 | 0.24 | .81 |
| Internalizing – Adult Academic Skills | 0.50 | .62 | 0.94 | .35 |
|  |  |  |  |  |
| Mother-Reported Outcomes |  |  |  |  |
| Social Competence – Adult Academic Skills | 0.00 | 1.00 | 0.24 | .81 |
| Externalizing – Adult Academic Skills | -1.72 | .09 | -0.92 | .36 |
| Internalizing – Adult Academic Skills | -1.37 | .17 | -0.88 | .38 |
|  |  |  |  |  |
| Self-Reported Outcomes |  |  |  |  |
| Social Competence – Adult Academic Skills | 0.00 | 1.00 | 0.23 | .82 |
| Externalizing – Adult Academic Skills | 0.43 | .66 | 0.66 | .51 |
| Internalizing – Adult Academic Skills | -0.65 | .51 | -0.22 | .82 |
|  |  |  |  |  |

*Note:* Bivariate associations = No covariates partialed from associations. Partial associations = Effects of demographics partialed from associations. Covariates include child sex, child race/ethnicity, family income-to-needs, and maternal education. Positive Z value indicates the left variable was larger. Negative Z value indicates the right variable was larger. The adult academic achievement variable contains the childhood objective measures of academic achievement used in the main analyses plus the *z*-standardized self-report of educational attainment in adulthood at 26 years of age.

**Supplementary Table 12**

Correlations between all study variables and adult social competence in the MLSRA

|  | Bivariate Associations  (*n* = 168) | Partial Associations  (*n* = 159) |
| --- | --- | --- |
| Proportion of Times Secure | **.26** | **.18** |
| Infant Attachment, 12mo | **.24** | .15 |
| Infant Attachment, 18mo | **.19** | .14 |
| Teacher-Rated Externalizing | **-.49** | **-.42** |
| Teacher-Rated Internalizing | **-.64** | **-.64** |
| Mother-Rated Externalizing | **-.38** | **-.30** |
| Mother-Rated Internalizing | **-.32** | **-.31** |
| Self-Rated Externalizing | **-.35** | **-.27** |
| Self-Rated Internalizing | **-.23** | **-.29** |
| Objective Academic Skills | **.45** | **.37** |

*Note.* Adult social competence contains the childhood teacher-reported social competence used in main analysis as well as the *z*-standardized objectively measured relationship effectiveness of engagement variable assessed at ages 23 and 26. Covariates include child sex, child race/ethnicity, family socioeconomic status, and maternal education. Bolded values indicate *p* < .05.

**Supplementary Table 13**

Steiger’s Z comparisons of associations between infant attachment, adult social competence, and academic skills for the MLSRA

|  | Bivariate associations  (*n* = 168) | | Partial associations  (*n* = 159) | |
| --- | --- | --- | --- | --- |
| Comparisons | *Z* | *p* | *Z* | *p* |
| Teacher-Reported Outcomes |  |  |  |  |
| Social Competence – Externalizing | -2.61 | .01 | -1.65 | .10 |
| Social Competence – Internalizing | -2.18 | .03 | -1.49 | .14 |
| Social Competence – Objective Academic Skills | 1.18 | .24 | 1.14 | .26 |

*Note:* Bivariate associations = No covariates partialed from associations. Partial associations = Effects of demographics partialed from associations. Covariates include child sex, child race/ethnicity, family socioeconomic status, and maternal education. Positive Z value indicates the left variable was larger. Negative Z value indicates the right variable was larger. Adult social competence contains the childhood teacher-reported social competence used in main analysis as well as the *z*-standardized objectively measured relationship effectiveness of engagement assessed at ages 23 and 26.

**Supplementary Table 14**

Correlations between all study variables and the 15-month Strange Situation Procedure with ‘cannot classify’ cases discarded in the SECCYD

|  | Bivariate associations  (*n* = 869) | Partial associations  (*n* = 868) |
| --- | --- | --- |
| Teacher-Rated Social Competence | **.06** | .02 |
| Teacher-Rated Externalizing | **-.06** | -.05 |
| Teacher-Rated Internalizing | **-.12** | **-.11** |
| Mother-Rated Social Competence | **.09** | .05 |
| Mother-Rated Externalizing | -.01 | .01 |
| Mother-Rated Internalizing | -.02 | .00 |
| Self-Rated Social Competence | **.08** | **.07** |
| Self-Rated Externalizing | **-.08** | **-.08** |
| Self-Rated Internalizing | -.04 | -.04 |
| Objective Academic Skills | **.08** | .02 |

*Note.*  Bivariate associations = No covariates partialed from associations. Partial associations = Effects of demographics partialed from associations. Covariates include child sex, child ethnicity, family income-to-needs, and maternal education. Bolded values indicate *p* < .05.

**Supplementary Table 15** Steiger’s Z comparisons of associations between 15-month Strange Situation Procedure with ‘cannot classify’ cases discarded, socioemotional outcomes, and academic skills for the SECCYD

|  | Bivariate associations  (*n* = 869) | | Partial associations  (*n* = 868) | |
| --- | --- | --- | --- | --- |
| Comparisons | *Z* | *p* | *Z* | *p* |
| Teacher-Reported Outcomes |  |  |  |  |
| Social Competence – Objective Academic Skills | -0.29 | .77 | -0.25 | .80 |
| Social Competence – Teacher Academic Skills | 0.00 | 1.00 | 0.00 | 1.00 |
| Social Competence – Externalizing | -0.75 | .45 | -1.04 | .30 |
| Social Competence – Internalizing | -1.94 | .05 | -2.80 | .01 |
| Externalizing – Internalizing | 1.00 | .32 | -1.45 | .15 |
| Externalizing – Objective Academic Skills | 0.25 | .80 | 0.46 | .64 |
| Externalizing – Teacher Academic Skills | 0.55 | .58 | 0.77 | .44 |
| Internalizing – Objective Academic Skills | 1.21 | .23 | 1.83 | .07 |
| Internalizing – Teacher Academic Skills | 1.61 | .11 | 2.31 | .02 |
| Mother-Reported Outcomes |  |  |  |  |
| Social Competence – Objective Academic Skills | 0.25 | .81 | 0.45 | .65 |
| Social Competence – Teacher Academic Skills | 0.50 | .62 | 0.70 | .49 |
| Social Competence – Externalizing | 1.84 | .07 | 1.49 | .14 |
| Social Competence – Internalizing | 1.95 | .05 | 1.38 | .17 |
| Externalizing – Internalizing | 0.39 | .70 | 0.00 | 1.00 |
| Externalizing – Objective Academic Skills | -1.19 | .24 | -0.67 | .51 |
| Externalizing – Teacher Academic Skills | -0.96 | .34 | -0.46 | .65 |
| Internalizing – Objective Academic Skills | -1.33 | .18 | -0.64 | .52 |
| Internalizing – Teacher Academic Skills | -1.12 | .26 | -.0.43 | .66 |
| Self-Reported Outcomes |  |  |  |  |
| Social Competence – Objective Academic Skills | 0.47 | .64 | 0.89 | .37 |
| Social Competence – Teacher Academic Skills | 0.72 | .47 | 1.14 | .25 |
| Social Competence – Externalizing | -0.29 | .77 | -0.28 | .78 |
| Social Competence – Internalizing | 1.04 | .30 | 0.78 | .43 |
| Externalizing – Internalizing | 1.57 | .12 | 1.26 | .21 |
| Externalizing – Objective Academic Skills | 0.63 | .53 | 1.11 | .27 |
| Externalizing – Teacher Academic Skills | 0.87 | .38 | 1.26 | .21 |
| Internalizing – Objective Academic Skills | -0.43 | .67 | 0.22 | .83 |
| Internalizing – Teacher Academic Skills | -0.21 | .83 | 0.43 | .67 |
| *Note:* Bivariate associations = No covariates partialed from associations. Partial associations = Effects of demographics partialed from associations. Covariates include child sex, child ethnicity, family income-to-needs, and maternal education. Positive Z value indicates the left variable was larger. Negative Z value indicates the right variable was larger | | | | |

**Supplementary Table 16** Path coefficients and indirect effects for mediation models with proportion of times secure as independent variable, demographic variables as mediators, and socioemotional and academic outcomes as dependent variables in the MLSRA

|  | Path A | | | Path B | | | Path C’ | | | Total Effect | | | Indirect Effects | | | I/T |
| --- | --- | --- | --- | --- | --- | --- | --- | --- | --- | --- | --- | --- | --- | --- | --- | --- |
|  |  |  |  |  |  |  |  |  |  |  | *95% Credible Interval* | |  | *95% Credible Interval* | |  |
|  | *B* | *pSD* | *p* | *B* | *pSD* | *p* | *B* | *pSD* | *p* | *B* | *Lower* | *Upper* | *B* | *Lower* | *Upper* |  |
| **Teacher-Reported Outcomes** | | | | | | | | | | | | | | | |  |
| Social Competence |  |  |  |  |  |  | 0.09 | 0.07 | .109 | 0.23 | 0.09 | 0.35 | 0.09 | 0.03 | 0.17 | 39% |
| Birth sex | 0.08 | 0.08 | .150 | 0.27 | 0.08 | .001 |  |  |  |  |  |  | 0.02 | -0.02 | 0.08 |  |
| Race/ethnicity | 0.09 | 0.08 | .140 | 0.10 | 0.09 | .148 |  |  |  |  |  |  | 0.01 | -0.01 | 0.04 |  |
| Maternal education | 0.21 | 0.06 | .001 | 0.29 | 0.08 | < .001 |  |  |  |  |  |  | 0.06 | 0.02 | 0.12 |  |
| Socioeconomic index | 0.16 | 0.07 | .009 | 0.02 | 0.08 | .419 |  |  |  |  |  |  | 0.00 | -0.03 | 0.03 |  |
| Externalizing |  |  |  |  |  |  | 0.03 | 0.07 | .347 | -0.05 | 0.07 | 0.26 | -0.07 | -0.16 | 0.09 | N/A* |
| Birth sex | 0.08 | 0.09 | .193 | -0.03 | 0.09 | .363 |  |  |  |  |  |  | 0.00 | -0.03 | 0.02 |  |
| Race/ethnicity | 0.08 | 0.09 | .162 | -0.34 | 0.09 | < .001 |  |  |  |  |  |  | -0.03 | -0.10 | 0.03 |  |
| Maternal education | 0.21 | 0.06 | .002 | -0.24 | 0.08 | .002 |  |  |  |  |  |  | -0.05 | -0.10 | -0.01 |  |
| Socioeconomic index | 0.16 | 0.07 | .013 | 0.04 | 0.08 | .295 |  |  |  |  |  |  | 0.01 | -0.03 | 0.04 |  |
| Internalizing |  |  |  |  |  |  | -0.11 | 0.07 | .058 | -0.15 | -0.28 | -0.01 | -0.04 | -0.10 | 0.01 | 27% |
| Birth sex | 0.08 | 0.09 | .209 | -0.08 | 0.09 | .188 |  |  |  |  |  |  | 0.00 | -0.04 | 0.02 |  |
| Race/ethnicity | 0.09 | 0.09 | .145 | -0.12 | 0.10 | .126 |  |  |  |  |  |  | -0.01 | -0.05 | 0.01 |  |
| Maternal education | 0.21 | 0.06 | .002 | -0.15 | 0.08 | .023 |  |  |  |  |  |  | -0.03 | -0.08 | 0.00 |  |
| Socioeconomic index | 0.16 | 0.07 | .013 | 0.07 | 0.08 | .205 |  |  |  |  |  |  | 0.01 | -0.02 | 0.05 |  |
| **Mother-Reported Outcomes** | | | | | | | | | | | | | | | |  |
| Externalizing |  |  |  |  |  |  | -0.08 | 0.07 | .131 | -0.12 | -0.26 | 0.01 | -0.05 | -0.11 | 0.01 | 71% |
| Birth sex | 0.08 | 0.09 | .189 | 0.04 | 0.09 | .343 |  |  |  |  |  |  | 0.00 | -0.02 | 0.03 |  |
| Race/ethnicity | 0.10 | 0.08 | .119 | 0.08 | 0.10 | .218 |  |  |  |  |  |  | 0.00 | -0.02 | 0.04 |  |
| Maternal education | 0.21 | 0.06 | .001 | -0.32 | 0.08 | < .001 |  |  |  |  |  |  | -0.07 | -0.12 | -0.02 |  |
| Socioeconomic index | 0.16 | 0.07 | .015 | 0.06 | 0.08 | .251 |  |  |  |  |  |  | 0.01 | -0.02 | 0.04 |  |
| Internalizing |  |  |  |  |  |  | -0.12 | 0.07 | .037 | -0.14 | -0.27 | -0.00 | -0.02 | -0.07 | 0.04 | 14% |
| Birth sex | 0.08 | 0.09 | .182 | 0.10 | 0.09 | .161 |  |  |  |  |  |  | 0.00 | -0.01 | 0.04 |  |
| Race/ethnicity | 0.09 | 0.08 | .117 | 0.05 | 0.10 | .311 |  |  |  |  |  |  | 0.00 | -0.02 | 0.03 |  |
| Maternal education | 0.21 | 0.06 | .001 | -0.15 | 0.08 | .032 |  |  |  |  |  |  | -0.03 | -0.08 | 0.00 |  |
| Socioeconomic index | 0.16 | 0.07 | .015 | 0.02 | 0.09 | .408 |  |  |  |  |  |  | 0.00 | -0.03 | 0.04 |  |
| **Self-Reported Outcomes** |  |  |  |  |  |  |  |  |  |  |  |  |  |  |  |  |
| Externalizing |  |  |  |  |  |  | -0.07 | 0.07 | .158 | -0.14 | -0.28 | 0.00 | -0.06 | -0.13 | -0.01 | 43% |
| Birth sex | 0.08 | 0.08 | .190 | -0.11 | 0.09 | .119 |  |  |  |  |  |  | -0.01 | -0.04 | 0.01 |  |
| Race/ethnicity | 0.08 | 0.08 | .158 | 0.00 | 0.10 | .494 |  |  |  |  |  |  | 0.00 | -0.03 | 0.03 |  |
| Maternal education | 0.21 | 0.06 | .001 | -0.22 | 0.08 | .005 |  |  |  |  |  |  | -0.04 | -0.10 | -0.01 |  |
| Socioeconomic index | 0.16 | 0.07 | .012 | -0.06 | 0.08 | .230 |  |  |  |  |  |  | -0.01 | -0.04 | 0.02 |  |
| Internalizing |  |  |  |  |  |  | -0.13 | 0.07 | .045 | -0.12 | -0.26 | 0.02 | 0.00 | -0.05 | 0.06 | 0% |
| Birth sex | 0.08 | 0.08 | .175 | 0.02 | 0.10 | .410 |  |  |  |  |  |  | 0.00 | -0.02 | 0.03 |  |
| Race/ethnicity | 0.08 | 0.08 | .169 | 0.04 | 0.10 | .373 |  |  |  |  |  |  | 0.00 | -0.02 | 0.03 |  |
| Maternal education | 0.21 | 0.06 | .001 | 0.02 | 0.08 | .424 |  |  |  |  |  |  | 0.00 | -0.03 | 0.04 |  |
| Socioeconomic index | 0.16 | 0.07 | .012 | -0.02 | 0.08 | .418 |  |  |  |  |  |  | 0.00 | -0.03 | 0.03 |  |
| **Objectively Measured Outcomes** | |  |  |  |  |  |  |  |  |  |  |  |  |  |  |  |
| Academic Skills |  |  |  |  |  |  | 0.21 | 0.06 | .001 | 0.32 | 0.19 | 0.43 | 0.11 | 0.04 | 0.19 | 34% |
| Birth sex | 0.07 | 0.09 | .222 | 0.17 | 0.08 | .027 |  |  |  |  |  |  | 0.01 | -0.02 | 0.05 |  |
| Race/ethnicity | 0.09 | 0.08 | .129 | 0.22 | 0.08 | .003 |  |  |  |  |  |  | 0.02 | -0.02 | 0.07 |  |
| Maternal education | 0.21 | 0.06 | .001 | 0.30 | 0.07 | < .001 |  |  |  |  |  |  | 0.06 | 0.02 | 0.12 |  |
| Socioeconomic index | 0.16 | 0.07 | .013 | 0.08 | 0.07 | .166 |  |  |  |  |  |  | 0.01 | -0.01 | 0.04 |  |

*Note. n* = 220. *B* = standardized beta coefficient estimate; *pSD* = posterior standard deviation; *p* = one-tailed *p*-value. Child sex was coded as 1 = male, 2 = female. Race/ethnicity was coded as 1 = White/non-Hispanic, 0 = non-White or Hispanic. I/T = indirect effect/total effect. *As described by Wen & Fan (2015), in mediation models where the direct effect (C') and the indirect effect have opposite signs, the I/T statistic is not calculable or interpretable because the indirect effect could be any value and has no theoretical bounds.

**Supplementary Table 17**

Path coefficients and indirect effects for mediation models with 12-month Strange Situation Procedure as independent variable, demographic variables as mediators, and socioemotional and academic outcomes as dependent variables in the MLSRA

|  | Path A | | | Path B | | | Path C’ | | | Total Effect | | | Indirect Effects | | | I/T |
| --- | --- | --- | --- | --- | --- | --- | --- | --- | --- | --- | --- | --- | --- | --- | --- | --- |
|  |  |  |  |  |  |  |  |  |  |  | *95% Credible Interval* | |  | *95% Credible Interval* | |  |
|  | *B* | *pSD* | *p* | *B* | *pSD* | *p* | *B* | *pSD* | *p* | *B* | *Lower* | *Upper* | *B* | *Lower* | *Upper* |  |
| **Teacher-Reported Outcomes** | | | | | | | | | | | | | | | |  |
| Social Competence |  |  |  |  |  |  | 0.10 | 0.07 | .066 | 0.20 | 0.06 | 0.32 | 0.09 | 0.02 | 0.17 | 45% |
| Birth sex | 0.11 | 0.08 | .113 | 0.25 | 0.08 | .003 |  |  |  |  |  |  | 0.02 | -0.02 | 0.07 |  |
| Race/ethnicity | 0.03 | 0.09 | .380 | 0.11 | 0.09 | .119 |  |  |  |  |  |  | 0.00 | -0.02 | 0.04 |  |
| Maternal education | 0.22 | 0.07 | .001 | 0.29 | 0.08 | < .001 |  |  |  |  |  |  | 0.06 | 0.02 | 0.12 |  |
| Socioeconomic index | 0.10 | 0.07 | .083 | 0.01 | 0.08 | .472 |  |  |  |  |  |  | 0.00 | -0.02 | 0.02 |  |
| Externalizing |  |  |  |  |  |  | 0.03 | 0.07 | .324 | -0.02 | -0.16 | 0.12 | -0.05 | -0.13 | 0.02 | N/A* |
| Birth sex | 0.07 | 0.08 | .156 | -0.01 | 0.09 | .455 |  |  |  |  |  |  | 0.00 | -0.02 | 0.02 |  |
| Race/ethnicity | 0.02 | 0.08 | .417 | -0.35 | 0.09 | .001 |  |  |  |  |  |  | -0.01 | -0.07 | 0.05 |  |
| Maternal education | 0.22 | 0.06 | < .001 | -0.24 | 0.08 | .001 |  |  |  |  |  |  | -0.05 | -0.11 | -0.01 |  |
| Socioeconomic index | 0.10 | 0.07 | .077 | 0.06 | 0.09 | .255 |  |  |  |  |  |  | 0.00 | -0.02 | 0.03 |  |
| Internalizing |  |  |  |  |  |  | -0.11 | 0.07 | .075 | -0.14 | -0.28 | -0.001 | -0.04 | -0.10 | 0.01 | 29% |
| Birth sex | 0.08 | 0.08 | .155 | -0.07 | 0.09 | .247 |  |  |  |  |  |  | 0.00 | -0.03 | 0.01 |  |
| Race/ethnicity | 0.03 | 0.09 | .386 | -0.12 | 0.10 | .090 |  |  |  |  |  |  | 0.00 | -0.04 | 0.02 |  |
| Maternal education | 0.23 | 0.06 | < .001 | -0.17 | 0.08 | .017 |  |  |  |  |  |  | -0.04 | -0.08 | 0.00 |  |
| Socioeconomic index | 0.11 | 0.07 | .075 | 0.08 | 0.08 | .189 |  |  |  |  |  |  | 0.01 | -0.01 | 0.04 |  |
| **Mother-Reported Outcomes** | | | | | | | | | | | | | | | | |
| Externalizing |  |  |  |  |  |  | -0.02 | 0.07 | .377 | -0.09 | -0.23 | 0.06 | -0.06 | -0.13 | -0.01 | 67% |
| Birth sex | 0.10 | 0.08 | .108 | 0.04 | 0.09 | .377 |  |  |  |  |  |  | 0.00 | -0.02 | 0.03 |  |
| Race/ethnicity | 0.03 | 0.08 | .343 | 0.01 | 0.10 | .463 |  |  |  |  |  |  | 0.00 | -0.02 | 0.02 |  |
| Maternal education | 0.22 | 0.07 | .002 | -0.34 | 0.08 | < .001 |  |  |  |  |  |  | -0.07 | -0.14 | -0.02 |  |
| Socioeconomic index | 0.10 | 0.07 | .078 | 0.07 | 0.09 | .221 |  |  |  |  |  |  | 0.00 | -0.01 | 0.04 |  |
| Internalizing |  |  |  |  |  |  | -0.10 | 0.08 | .080 | -0.13 | -0.27 | 0.01 | -0.02 | -0.08 | 0.02 | 15% |
| Birth sex | 0.10 | 0.08 | .106 | 0.09 | 0.09 | .162 |  |  |  |  |  |  | 0.01 | -0.01 | 0.04 |  |
| Race/ethnicity | 0.03 | 0.08 | .335 | 0.01 | 0.10 | .454 |  |  |  |  |  |  | 0.00 | -0.02 | 0.02 |  |
| Maternal education | 0.22 | 0.07 | .002 | -0.16 | 0.08 | .024 |  |  |  |  |  |  | -0.03 | -0.08 | 0.00 |  |
| Socioeconomic index | 0.10 | 0.07 | .079 | 0.01 | 0.09 | .452 |  |  |  |  |  |  | 0.00 | -0.02 | 0.03 |  |
| **Self-Reported Outcomes** |  |  |  |  |  |  |  |  |  |  |  |  |  |  |  |  |
| Externalizing |  |  |  |  |  |  | -0.05 | 0.07 | .249 | -0.11 | -0.25 | 0.03 | -0.06 | -0.12 | -0.01 | 55% |
| Birth sex | 0.10 | 0.08 | .122 | -0.09 | 0.09 | .189 |  |  |  |  |  |  | -0.01 | -0.04 | 0.01 |  |
| Race/ethnicity | 0.02 | 0.08 | .374 | 0.00 | 0.10 | .493 |  |  |  |  |  |  | 0.00 | -0.02 | 0.02 |  |
| Maternal education | 0.22 | 0.06 | < .001 | -0.23 | 0.08 | .005 |  |  |  |  |  |  | -0.05 | -0.10 | -0.01 |  |
| Socioeconomic index | 0.10 | 0.07 | .080 | -0.07 | 0.08 | .201 |  |  |  |  |  |  | -0.01 | -0.04 | 0.01 |  |
| Internalizing |  |  |  |  |  |  | -0.06 | 0.08 | .213 | -0.06 | -0.21 | 0.08 | 0.00 | -0.05 | 0.05 | 0% |
| Birth sex | 0.09 | 0.08 | .141 | 0.03 | 0.10 | .382 |  |  |  |  |  |  | 0.00 | -0.02 | 0.03 |  |
| Race/ethnicity | 0.02 | 0.07 | .394 | 0.02 | 0.10 | .407 |  |  |  |  |  |  | 0.00 | -0.02 | 0.02 |  |
| Maternal education | 0.22 | 0.06 | < .001 | -0.01 | 0.08 | .454 |  |  |  |  |  |  | 0.00 | -0.04 | 0.04 |  |
| Socioeconomic index | 0.10 | 0.07 | .082 | -0.02 | 0.08 | .391 |  |  |  |  |  |  | 0.00 | -0.03 | 0.02 |  |
| **Objectively Measured Outcomes** | |  |  |  |  |  |  |  |  |  |  |  |  |  |  |  |
| Academic Skills |  |  |  |  |  |  | 0.17 | 0.06 | .007 | 0.26 | 0.13 | 0.38 | 0.10 | 0.02 | 0.18 | 38% |
| Birth sex | 0.07 | 0.07 | .176 | 0.10 | 0.08 | .113 |  |  |  |  |  |  | 0.00 | -0.01 | 0.03 |  |
| Race/ethnicity | 0.05 | 0.09 | .306 | 0.29 | 0.08 | < .001 |  |  |  |  |  |  | 0.01 | -0.05 | 0.07 |  |
| Maternal education | 0.22 | 0.06 | < .001 | 0.31 | 0.07 | < .001 |  |  |  |  |  |  | 0.07 | 0.02 | 0.13 |  |
| Socioeconomic index | 0.11 | 0.07 | .073 | 0.09 | 0.08 | .122 |  |  |  |  |  |  | 0.01 | -0.01 | 0.04 |  |

*Note. n* = 212. *B* = standardized beta coefficient estimate; *pSD* = posterior standard deviation; *p* = one-tailed *p*-value. Child sex was coded as 1 = male, 2 = female. Race/ethnicity was coded as 1 = White/non-Hispanic, 0 = non-White or Hispanic. I/T = indirect effect/total effect. *As described by Wen & Fan (2015), in mediation models where the direct effect (C') and the indirect effect have opposite signs, the I/T statistic is not calculable or interpretable because the indirect effect could be any value and has no theoretical bounds.

**Supplementary Table 18**

Path coefficients and indirect effects for mediation models with 18-month Strange Situation Procedure as independent variable, demographic variables as mediators, and socioemotional and academic outcomes as dependent variables in the MLSRA

|  | Path A | | | Path B | | | Path C’ | | | Total Effects | | | Indirect Effects | | | I/T |
| --- | --- | --- | --- | --- | --- | --- | --- | --- | --- | --- | --- | --- | --- | --- | --- | --- |
|  |  |  |  |  |  |  |  |  |  |  | *95% Credible Interval* | |  | *95% Credible Interval* | |  |
|  | *B* | *pSD* | *p* | *B* | *pSD* | *p* | *B* | *pSD* | *p* | *B* | *Lower* | *Upper* | *B* | *Lower* | *Upper* |  |
| **Teacher-Reported Outcomes** | | | | | | | | | | | | | | | |  |
| Social Competence |  |  |  |  |  |  | 0.04 | 0.07 | .291 | 0.11 | -0.02 | 0.26 | 0.07 | 0.00 | 0.16 | 64% |
| Birth sex | 0.06 | 0.10 | .265 | 0.29 | 0.08 | .001 |  |  |  |  |  |  | 0.02 | -0.03 | 0.08 |  |
| Race/ethnicity | 0.13 | 0.10 | .084 | 0.11 | 0.10 | .137 |  |  |  |  |  |  | 0.01 | -0.01 | 0.06 |  |
| Maternal education | 0.12 | 0.07 | .033 | 0.35 | 0.08 | < .001 |  |  |  |  |  |  | 0.04 | 0.00 | 0.10 |  |
| Socioeconomic index | 0.16 | 0.07 | .014 | 0.01 | 0.08 | .463 |  |  |  |  |  |  | 0.00 | -0.03 | 0.03 |  |
| Externalizing |  |  |  |  |  |  | 0.01 | 0.07 | .431 | -0.06 | -0.19 | 0.09 | -0.07 | -0.16 | 0.00 | N/A* |
| Birth sex | 0.07 | 0.09 | .235 | -0.05 | 0.09 | .285 |  |  |  |  |  |  | 0.00 | -0.03 | 0.02 |  |
| Race/ethnicity | 0.12 | 0.09 | .076 | -0.36 | 0.09 | < .001 |  |  |  |  |  |  | -0.04 | -0.12 | 0.02 |  |
| Maternal education | 0.12 | 0.07 | .035 | -0.27 | 0.08 | .001 |  |  |  |  |  |  | -0.03 | -0.08 | 0.00 |  |
| Socioeconomic index | 0.16 | 0.07 | .015 | 0.05 | 0.08 | .268 |  |  |  |  |  |  | 0.01 | -0.02 | 0.04 |  |
| Internalizing |  |  |  |  |  |  | -0.07 | 0.07 | .176 | -0.11 | -0.24 | 0.03 | -0.04 | -0.24 | 0.03 | N/A* |
| Birth sex | 0.07 | 0.09 | .245 | -0.14 | 0.09 | .056 |  |  |  |  |  |  | -0.01 | -0.05 | 0.01 |  |
| Race/ethnicity | 0.14 | 0.09 | .074 | -0.14 | 0.10 | .077 |  |  |  |  |  |  | -0.04 | -0.07 | 0.01 |  |
| Maternal education | 0.12 | 0.07 | .035 | -0.22 | 0.08 | .004 |  |  |  |  |  |  | -0.02 | -0.07 | 0.00 |  |
| Socioeconomic index | 0.16 | 0.07 | .016 | 0.06 | 0.08 | .219 |  |  |  |  |  |  | 0.01 | -0.02 | 0.04 |  |
| **Mother-Reported Outcomes** |  |  |  |  |  |  |  |  |  |  |  |  |  |  |  |  |
| Externalizing |  |  |  |  |  |  | -0.10 | 0.07 | .090 | -0.12 | -0.26 | 0.03 | -0.02 | -0.08 | 0.04 | 17% |
| Birth sex | 0.06 | 0.08 | .220 | 0.02 | 0.09 | .405 |  |  |  |  |  |  | 0.00 | -0.02 | 0.03 |  |
| Race/ethnicity | 0.15 | 0.09 | .053 | 0.07 | 0.10 | .260 |  |  |  |  |  |  | 0.01 | -0.02 | 0.06 |  |
| Maternal education | 0.12 | 0.07 | .034 | -0.33 | 0.08 | < .001 |  |  |  |  |  |  | -0.04 | -0.09 | 0.00 |  |
| Socioeconomic index | 0.16 | 0.07 | .015 | 0.06 | 0.08 | .230 |  |  |  |  |  |  | 0.01 | -0.02 | 0.05 |  |
| Internalizing |  |  |  |  |  |  | -0.09 | 0.07 | .116 | -0.10 | -0.24 | 0.04 | -0.01 | -0.07 | 0.04 | N/A* |
| Birth sex | 0.06 | 0.09 | .274 | 0.09 | 0.09 | .172 |  |  |  |  |  |  | 0.00 | -0.02 | 0.04 |  |
| Race/ethnicity | 0.12 | 0.09 | .089 | 0.02 | 0.10 | .424 |  |  |  |  |  |  | 0.00 | -0.03 | 0.04 |  |
| Maternal education | 0.12 | 0.07 | .032 | -0.18 | 0.08 | .015 |  |  |  |  |  |  | -0.02 | -0.06 | 0.00 |  |
| Socioeconomic index | 0.16 | 0.07 | .014 | 0.02 | 0.09 | .417 |  |  |  |  |  |  | 0.00 | -0.03 | 0.04 |  |
| **Self-Reported Outcomes** |  |  |  |  |  |  |  |  |  |  |  |  |  |  |  |  |
| Externalizing |  |  |  |  |  |  | -0.08 | 0.08 | .167 | -0.12 | -0.27 | 0.03 | -0.04 | -0.12 | 0.01 | 33% |
| Birth sex | 0.04 | 0.08 | .301 | -0.15 | 0.09 | .062 |  |  |  |  |  |  | 0.00 | -0.04 | 0.02 |  |
| Race/ethnicity | 0.16 | 0.08 | .029 | -0.02 | 0.10 | .426 |  |  |  |  |  |  | 0.00 | -0.04 | 0.03 |  |
| Maternal education | 0.12 | 0.07 | .041 | -0.23 | 0.08 | .005 |  |  |  |  |  |  | -0.03 | -0.07 | 0.00 |  |
| Socioeconomic index | 0.16 | 0.07 | .014 | -0.05 | 0.08 | .249 |  |  |  |  |  |  | -0.01 | -0.05 | 0.02 |  |
| Internalizing |  |  |  |  |  |  | -0.16 | 0.08 | .024 | -0.15 | -0.29 | 0.01 | 0.01 | -0.04 | 0.07 | N/A* |
| Birth sex | 0.04 | 0.09 | .311 | 0.01 | 0.10 | .452 |  |  |  |  |  |  | 0.00 | -0.02 | 0.03 |  |
| Race/ethnicity | 0.16 | 0.08 | .040 | 0.06 | 0.10 | .287 |  |  |  |  |  |  | 0.01 | -0.03 | 0.05 |  |
| Maternal education | 0.12 | 0.07 | .040 | 0.03 | 0.09 | .374 |  |  |  |  |  |  | 0.00 | -0.02 | 0.03 |  |
| SEI | 0.16 | 0.07 | .013 | 0.00 | 0.09 | .499 |  |  |  |  |  |  | 0.00 | -0.03 | 0.03 |  |
| **Objectively Measured Outcomes** | |  |  |  |  |  |  |  |  |  |  |  |  |  |  |  |
| Academic Skills |  |  |  |  |  |  | 0.20 | 0.07 | .003 | 0.28 | 0.14 | 0.41 | 0.08 | 0.00 | 0.16 | 29% |
| Birth sex | 0.03 | 0.09 | .381 | 0.15 | 0.09 | .037 |  |  |  |  |  |  | 0.00 | -0.03 | 0.03 |  |
| Race/ethnicity | 0.12 | 0.10 | .100 | 0.21 | 0.09 | .008 |  |  |  |  |  |  | 0.02 | -0.02 | 0.08 |  |
| Maternal education | 0.13 | 0.07 | .040 | 0.35 | 0.07 | < .001 |  |  |  |  |  |  | 0.04 | -0.01 | 0.10 |  |
| Socioeconomic index | 0.17 | 0.07 | .013 | 0.06 | 0.08 | .208 |  |  |  |  |  |  | 0.01 | -0.02 | 0.04 |  |

*Note. n* = 197. *B* = standardized beta coefficient estimate; *pSD* = posterior standard deviation; *p* = one-tailed *p*-value. Child sex was coded as 1 = male, 2 = female. Race/ethnicity was coded as 1 = White/non-Hispanic, 0 = non-White or Hispanic. *As described by Wen & Fan (2015), in mediation models where the direct effect (C') and the indirect effect have opposite signs, the I/T statistic is not calculable or interpretable because the indirect effect could be any value and has no theoretical bounds.

**Supplementary Table 19**

Path coefficients and indirect effects for mediation models with Proportion of Times Secure as independent variable, demographic variables as mediators, and adult social competence and adult academic achievement as dependent variables in the MLSRA

|  | Path A | | | Path B | | | Path C’ | | | Total Effects | | | Indirect Effects | | | I/T |
| --- | --- | --- | --- | --- | --- | --- | --- | --- | --- | --- | --- | --- | --- | --- | --- | --- |
|  |  |  |  |  |  |  |  |  |  |  | *95% Credible Interval* | |  | *95% Credible Interval* | |  |
|  | *B* | *pSD* | *p* | *B* | *pSD* | *p* | *B* | *pSD* | *p* | *B* | *Lower* | *Upper* | *B* | *Lower* | *Upper* |  |
| Adult Social Competence |  |  |  |  |  |  | 0.14 | 0.07 | .022 | 0.23 | 0.09 | 0.35 | 0.09 | 0.03 | 0.16 | 39% |
| Birth sex | 0.08 | 0.08 | .161 | 0.24 | 0.08 | .003 |  |  |  |  |  |  | 0.02 | -0.02 | 0.07 |  |
| Race/ethnicity | 0.07 | 0.09 | .206 | 0.12 | 0.09 | .079 |  |  |  |  |  |  | 0.01 | -0.02 | 0.04 |  |
| Maternal education | 0.21 | 0.06 | .001 | 0.28 | 0.08 | < .001 |  |  |  |  |  |  | 0.06 | 0.02 | 0.11 |  |
| Socioeconomic index | 0.16 | 0.07 | .013 | 0.02 | 0.08 | .415 |  |  |  |  |  |  | 0.00 | -0.03 | 0.03 |  |
| Adult Academic Skills |  |  |  |  |  |  | 0.12 | 0.06 | .033 | 0.25 | 0.12 | 0.37 | 0.13 | 0.06 | 0.21 | 52% |
| Birth sex | 0.08 | 0.08 | .156 | 0.26 | 0.08 | < .001 |  |  |  |  |  |  | 0.02 | -0.02 | 0.07 |  |
| Race/ethnicity | 0.10 | 0.08 | .132 | 0.14 | 0.08 | .050 |  |  |  |  |  |  | 0.01 | -0.01 | 0.05 |  |
| Maternal education | 0.21 | 0.06 | .001 | 0.43 | 0.07 | < .001 |  |  |  |  |  |  | 0.09 | 0.04 | 0.16 |  |
| Socioeconomic index | 0.16 | 0.07 | .009 | 0.03 | 0.07 | .333 |  |  |  |  |  |  | 0.00 | -0.02 | 0.03 |  |

*Note. n* = 220. *B* = standardized beta coefficient estimate; *pSD* = posterior standard deviation; *p* = one-tailed *p*-value. Child sex was coded as 1 = male, 2 = female. Race/ethnicity was coded as 1 = White/non-Hispanic, 0 = non-White or Hispanic. Adult social competence contains the childhood teacher-reported social competence used in main analysis as well as the *z*-standardized objectively measured relationship effectiveness of engagement variable assessed at ages 23 and 26. The adult academic achievement variable contains the childhood objective measures of academic achievement used in the main analyses as well as the *z*-standardized self-reports of educational attainment in adulthood from 23, 26, 28, 32, 34, 37, and 39 years.

**Supplementary Table 20**

Path coefficients and indirect effects for mediation models with 15-month Strange Situation Procedure as independent variable, demographic variables as mediators, and socioemotional and academic outcomes as dependent variables in the SECCYD

|  | Path A | | | Path B | | | Path C’ | | | Total Effects | | | Indirect Effects | | | I/T |
| --- | --- | --- | --- | --- | --- | --- | --- | --- | --- | --- | --- | --- | --- | --- | --- | --- |
|  |  |  |  |  |  |  |  |  |  |  | *95% Credible Interval* | |  | *95% Credible Interval* | |  |
|  | *B* | *pSD* | *p* | *B* | *pSD* | *p* | *B* | *pSD* | *p* | *B* | *Lower* | *Upper* | *B* | *Lower* | *Upper* |  |
| **Teacher-Reported Outcomes** | | | | | | | | | | | | | | | |  |
| Social Competence |  |  |  |  |  |  | 0.02 | 0.03 | .283 | 0.05 | -0.01 | 0.11 | 0.03 | 0.01 | 0.06 | 60% |
| Birth sex | 0.04 | 0.04 | .165 | 0.00 | 0.04 | .449 |  |  |  |  |  |  | 0.00 | -0.01 | 0.00 |  |
| Race/ethnicity | -0.08 | 0.04 | .024 | -0.23 | 0.04 | < .001 |  |  |  |  |  |  | 0.02 | 0.00 | 0.04 |  |
| Maternal education | 0.07 | 0.03 | .012 | 0.23 | 0.04 | < .001 |  |  |  |  |  |  | 0.01 | 0.00 | 0.03 |  |
| Socioeconomic index | 0.04 | 0.03 | .065 | 0.06 | 0.04 | .043 |  |  |  |  |  |  | 0.00 | 0.00 | 0.01 |  |
| Externalizing |  |  |  |  |  |  | -0.02 | 0.03 | .282 | -0.05 | -0.10 | 0.01 | -0.03 | -0.05 | -0.01 | 60% |
| Birth sex | 0.03 | 0.04 | .176 | -0.02 | 0.04 | .315 |  |  |  |  |  |  | 0.00 | -0.01 | 0.00 |  |
| Race/ethnicity | -0.07 | 0.04 | .036 | 0.24 | 0.04 | < .001 |  |  |  |  |  |  | -0.02 | -0.04 | 0.00 |  |
| Maternal education | 0.06 | 0.03 | .010 | -0.16 | 0.04 | < .001 |  |  |  |  |  |  | -0.01 | -0.02 | 0.00 |  |
| Socioeconomic index | 0.04 | 0.03 | .068 | -0.02 | 0.04 | .275 |  |  |  |  |  |  | 0.00 | -0.01 | 0.00 |  |
| Internalizing |  |  |  |  |  |  | -0.08 | 0.03 | .002 | -0.10 | -0.16 | -0.04 | -0.02 | -0.03 | 0.00 | N/A* |
| Birth sex | 0.04 | 0.04 | .150 | 0.03 | 0.04 | .244 |  |  |  |  |  |  | 0.00 | 0.00 | 0.01 |  |
| Race/ethnicity | -0.07 | 0.04 | .027 | 0.06 | 0.05 | .108 |  |  |  |  |  |  | 0.00 | -0.02 | 0.00 |  |
| Maternal education | 0.06 | 0.03 | .010 | -0.16 | 0.04 | < .001 |  |  |  |  |  |  | -0.01 | -0.02 | 0.00 |  |
| Socioeconomic index | 0.04 | 0.03 | .068 | -0.05 | 0.04 | .095 |  |  |  |  |  |  | 0.00 | -0.01 | 0.00 |  |
| Academic Skills |  |  |  |  |  |  | 0.01 | 0.03 | .347 | 0.05 | -0.02 | 0.10 | 0.03 | 0.00 | 0.06 | 60% |
| Birth sex | 0.04 | 0.04 | .172 | -0.06 | 0.04 | .031 |  |  |  |  |  |  | 0.00 | -0.01 | 0.00 |  |
| Race/ethnicity | -0.07 | 0.04 | .058 | -0.20 | 0.04 | < .001 |  |  |  |  |  |  | 0.01 | 0.00 | 0.03 |  |
| Maternal education | 0.06 | 0.03 | .014 | 0.27 | 0.03 | < .001 |  |  |  |  |  |  | 0.02 | 0.00 | 0.03 |  |
| Socioeconomic index | 0.04 | 0.03 | .064 | 0.10 | 0.04 | .002 |  |  |  |  |  |  | 0.00 | 0.00 | 0.01 |  |
| **Mother-Reported Outcomes** |  |  |  |  |  |  |  |  |  |  |  |  |  |  |  |  |
| Social Competence |  |  |  |  |  |  | 0.06 | 0.03 | .022 | 0.09 | 0.03 | 0.15 | 0.03 | 0.00 | 0.06 | 33% |
| Birth sex | 0.03 | 0.04 | .191 | -0.09 | 0.04 | .006 |  |  |  |  |  |  | 0.00 | -0.01 | 0.00 |  |
| Race/ethnicity | -0.08 | 0.04 | .028 | -0.23 | 0.04 | < .001 |  |  |  |  |  |  | 0.02 | 0.00 | 0.04 |  |
| Maternal education | 0.07 | 0.03 | .011 | 0.19 | 0.03 | < .001 |  |  |  |  |  |  | 0.01 | 0.00 | 0.03 |  |
| Socioeconomic index | 0.05 | 0.03 | .051 | 0.05 | 0.04 | .098 |  |  |  |  |  |  | 0.00 | 0.00 | 0.01 |  |
| Externalizing |  |  |  |  |  |  | 0.01 | 0.03 | .355 | -0.004 | -0.06 | 0.05 | -0.02 | -0.03 | 0.00 | N/A* |
| Birth sex | 0.03 | 0.04 | .202 | 0.04 | 0.04 | .151 |  |  |  |  |  |  | 0.00 | 0.00 | 0.01 |  |
| Race/ethnicity | -0.07 | 0.04 | .025 | -0.03 | 0.04 | .303 |  |  |  |  |  |  | 0.00 | -0.01 | 0.01 |  |
| Maternal education | 0.07 | 0.03 | .011 | -0.22 | 0.03 | < .001 |  |  |  |  |  |  | -0.01 | -0.03 | 0.00 |  |
| Socioeconomic index | 0.04 | 0.03 | .065 | -0.08 | 0.03 | .008 |  |  |  |  |  |  | 0.00 | -0.01 | 0.00 |  |
| Internalizing |  |  |  |  |  |  | 0.00 | 0.03 | .487 | -0.01 | -0.07 | 0.05 | -0.01 | -0.02 | 0.00 | N/A* |
| Birth sex | 0.03 | 0.04 | .201 | 0.03 | 0.04 | .252 |  |  |  |  |  |  | 0.00 | 0.00 | 0.01 |  |
| Race/ethnicity | -0.07 | 0.04 | .029 | -0.03 | 0.05 | .271 |  |  |  |  |  |  | 0.00 | -0.01 | 0.01 |  |
| Maternal education | 0.07 | 0.03 | .011 | -0.15 | 0.03 | < .001 |  |  |  |  |  |  | -0.01 | -0.02 | 0.00 |  |
| Socioeconomic index | 0.04 | 0.03 | .064 | -0.06 | 0.04 | .051 |  |  |  |  |  |  | 0.00 | -0.01 | 0.00 |  |
|  |  |  |  |  |  |  |  |  |  |  |  |  |  |  |  |  |
| **Self-Reported Outcomes** |  |  |  |  |  |  |  |  |  |  |  |  |  |  |  |  |
| Social Competence |  |  |  |  |  |  | 0.05 | 0.03 | .056 | 0.07 | 0.00 | 0.13 | 0.02 | 0.00 | 0.03 | 29% |
| Birth sex | 0.03 | 0.04 | .201 | -0.02 | 0.04 | .282 |  |  |  |  |  |  | 0.00 | -0.01 | 0.00 |  |
| Race/ethnicity | -0.07 | 0.04 | .034 | -0.11 | 0.05 | .012 |  |  |  |  |  |  | 0.01 | 0.00 | 0.02 |  |
| Maternal education | 0.07 | 0.03 | .012 | 0.08 | 0.04 | .019 |  |  |  |  |  |  | 0.01 | 0.00 | 0.01 |  |
| Socioeconomic index | 0.04 | 0.03 | .072 | 0.09 | 0.04 | .018 |  |  |  |  |  |  | 0.00 | 0.00 | 0.01 |  |
| Externalizing |  |  |  |  |  |  | -0.06 | 0.03 | .033 | -0.08 | -0.14 | -0.01 | -0.01 | -0.03 | 0.00 | 13% |
| Birth sex | 0.04 | 0.04 | .172 | 0.04 | 0.04 | .139 |  |  |  |  |  |  | 0.00 | 0.00 | 0.01 |  |
| Race/ethnicity | -0.08 | 0.04 | .032 | 0.12 | 0.05 | .007 |  |  |  |  |  |  | -0.01 | -0.02 | 0.00 |  |
| Maternal education | 0.07 | 0.03 | .014 | -0.02 | 0.04 | .338 |  |  |  |  |  |  | 0.00 | -0.01 | 0.00 |  |
| Socioeconomic index | 0.05 | 0.03 | .068 | -0.07 | 0.04 | .047 |  |  |  |  |  |  | 0.00 | -0.01 | 0.00 |  |
| Internalizing |  |  |  |  |  |  | -0.04 | 0.03 | .145 | -0.08 | -0.20 | 0.06 | 0.00 | -0.01 | 0.01 | N/A* |
| Birth sex | 0.03 | 0.04 | .170 | 0.06 | 0.04 | .058 |  |  |  |  |  |  | 0.00 | 0.00 | 0.01 |  |
| Race/ethnicity | -0.08 | 0.04 | .027 | 0.04 | 0.05 | .241 |  |  |  |  |  |  | 0.00 | -0.01 | 0.01 |  |
| Maternal education | 0.07 | 0.03 | .013 | 0.00 | 0.04 | .488 |  |  |  |  |  |  | 0.00 | -0.01 | 0.01 |  |
| Socioeconomic index | 0.05 | 0.03 | .069 | -0.03 | 0.04 | .271 |  |  |  |  |  |  | 0.00 | -0.01 | 0.00 |  |
| **Objectively Measured Outcomes** | | | | | | | | | | | | | | | | |
| Academic Skills |  |  |  |  |  |  | 0.02 | 0.03 | .186 | 0.07 | 0.01 | 0.13 | 0.05 | 0.01 | 0.08 | 71% |
| Birth sex | 0.03 | 0.04 | .196 | 0.01 | 0.03 | .411 |  |  |  |  |  |  | 0.00 | 0.00 | 0.00 |  |
| Race/ethnicity | -0.07 | 0.04 | .062 | -0.27 | 0.04 | < .001 |  |  |  |  |  |  | 0.02 | 0.00 | 0.04 |  |
| Maternal education | 0.07 | 0.03 | .010 | 0.33 | 0.03 | < .001 |  |  |  |  |  |  | 0.02 | 0.00 | 0.04 |  |
| Socioeconomic index | 0.05 | 0.03 | .069 | 0.13 | 0.03 | < .001 |  |  |  |  |  |  | 0.01 | 0.00 | 0.02 |  |

*Note. n* = 1,191. *B* = standardized beta coefficient estimate; *pSD* = posterior standard deviation; *p* = one-tailed *p*-value. Child sex was coded as 1 = male, 2 = female. Race/ethnicity was coded as 1 = White/non-Hispanic, 0 = non-White or Hispanic. *As described by Wen & Fan (2015), in mediation models where the direct effect (C') and the indirect effect have opposite signs, the I/T statistic is not calculable or interpretable because the indirect effect could be any value and has no theoretical bounds.

**Supplementary Table 21**

Path coefficients and indirect effects for mediation models with proportion of times secure as independent variable, demographic variables as mediators, and socioemotional and academic outcomes as dependent variables in the SECCYD

|  | Path A | | | Path B | | | Path C’ | | | Direct Effect | | | Indirect Effects | | | I/T |
| --- | --- | --- | --- | --- | --- | --- | --- | --- | --- | --- | --- | --- | --- | --- | --- | --- |
|  |  |  |  |  |  |  |  |  |  |  | *95% Credible Interval* | |  | *95% Credible Interval* | |  |
|  | *B* | *pSD* | *p* | *B* | *pSD* | *p* | *B* | *pSD* | *p* | *B* | *Lower* | *Upper* | *B* | *Lower* | *Upper* |  |
| **Teacher-Reported Outcomes** | | | | | | | | | | | | | | | |  |
| Social Competence |  |  |  |  |  |  | 0.15 | 0.03 | < .001 | 0.23 | 0.17 | 0.28 | 0.08 | 0.06 | 0.11 | 35% |
| Birth sex | 0.05 | 0.03 | .078 | 0.00 | 0.04 | .463 |  |  |  |  |  |  | 0.00 | 0.00 | 0.01 |  |
| Race/ethnicity | -0.16 | 0.04 | < .001 | -0.20 | 0.04 | < .001 |  |  |  |  |  |  | 0.03 | 0.02 | 0.05 |  |
| Maternal education | 0.20 | 0.03 | < .001 | 0.22 | 0.03 | < .001 |  |  |  |  |  |  | 0.04 | 0.03 | 0.06 |  |
| Socioeconomic index | 0.14 | 0.03 | < .001 | 0.06 | 0.03 | .054 |  |  |  |  |  |  | 0.01 | 0.00 | 0.02 |  |
| Externalizing |  |  |  |  |  |  | -0.15 | 0.03 | < .001 | -0.21 | -0.27 | -0.15 | -0.06 | -0.09 | -0.04 | 29% |
| Birth sex | 0.05 | 0.03 | .082 | -0.02 | 0.04 | .282 |  |  |  |  |  |  | 0.00 | -0.01 | 0.00 |  |
| Race/ethnicity | -0.16 | 0.04 | < .001 | 0.22 | 0.04 | < .001 |  |  |  |  |  |  | -0.03 | -0.06 | -0.02 |  |
| Maternal education | 0.20 | 0.03 | < .001 | -0.14 | 0.04 | < .001 |  |  |  |  |  |  | -0.03 | -0.04 | -0.04 |  |
| Socioeconomic index | 0.14 | 0.03 | < .001 | -0.02 | 0.04 | .313 |  |  |  |  |  |  | 0.00 | -0.01 | 0.01 |  |
| Internalizing |  |  |  |  |  |  | -0.17 | 0.03 | < .001 | -0.21 | -0.27 | -0.15 | -0.04 | -0.06 | -0.02 | 19% |
| Birth sex | 0.05 | 0.03 | .078 | 0.04 | 0.04 | .146 |  |  |  |  |  |  | 0.00 | 0.00 | 0.01 |  |
| Race/ethnicity | -0.17 | 0.04 | < .001 | 0.04 | 0.04 | .149 |  |  |  |  |  |  | -0.01 | -0.02 | 0.01 |  |
| Maternal education | 0.20 | 0.03 | < .001 | -0.14 | 0.04 | < .001 |  |  |  |  |  |  | -0.03 | -0.05 | -0.01 |  |
| Socioeconomic index | 0.14 | 0.03 | < .001 | -0.04 | 0.04 | .120 |  |  |  |  |  |  | -0.01 | -0.01 | 0.00 |  |
| Academic Skills |  |  |  |  |  |  | 0.11 | 0.03 | < .001 | 0.20 | 0.15 | 0.26 | 0.09 | 0.06 | 0.12 | 45% |
| Birth sex | 0.05 | 0.03 | .098 | -0.05 | 0.03 | .065 |  |  |  |  |  |  | 0.00 | -0.01 | 0.00 |  |
| Race/ethnicity | -0.16 | 0.04 | < .001 | -0.18 | 0.04 | < .001 |  |  |  |  |  |  | 0.03 | 0.01 | 0.05 |  |
| Maternal education | 0.19 | 0.03 | < .001 | 0.26 | 0.03 | < .001 |  |  |  |  |  |  | 0.05 | 0.03 | 0.07 |  |
| Socioeconomic index | 0.14 | 0.03 | < .001 | 0.10 | 0.03 | .002 |  |  |  |  |  |  | 0.01 | 0.00 | 0.03 |  |
| **Mother-Reported Outcomes** |  |  |  |  |  |  |  |  |  |  |  |  |  |  |  |  |
| Social Competence |  |  |  |  |  |  | 0.14 | 0.03 | < .001 | 0.22 | 0.16 | 0.27 | 0.07 | 0.05 | 0.10 | 32% |
| Birth sex | 0.04 | 0.04 | .156 | -0.09 | 0.04 | .007 |  |  |  |  |  |  | 0.00 | -0.01 | 0.00 |  |
| Race/ethnicity | -0.16 | 0.04 | < .001 | -0.21 | 0.04 | < .001 |  |  |  |  |  |  | 0.03 | 0.01 | 0.05 |  |
| Maternal education | 0.20 | 0.04 | < .001 | 0.18 | 0.03 | < .001 |  |  |  |  |  |  | 0.04 | 0.02 | 0.05 |  |
| Socioeconomic index | 0.14 | 0.03 | < .001 | 0.05 | 0.04 | .071 |  |  |  |  |  |  | 0.01 | 0.00 | 0.02 |  |
| Externalizing |  |  |  |  |  |  | -0.10 | 0.03 | .001 | -0.15 | -0.20 | -0.09 | -0.05 | -0.07 | -0.03 | 33% |
| Birth sex | 0.04 | 0.04 | .129 | 0.05 | 0.04 | .102 |  |  |  |  |  |  | 0.00 | 0.00 | 0.01 |  |
| Race/ethnicity | -0.16 | 0.04 | < .001 | -0.04 | 0.04 | .198 |  |  |  |  |  |  | 0.01 | -0.01 | 0.02 |  |
| Maternal education | 0.20 | 0.03 | < .001 | -0.21 | 0.03 | < .001 |  |  |  |  |  |  | -0.04 | -0.06 | -0.03 |  |
| Socioeconomic index | 0.14 | 0.03 | < .001 | -0.09 | 0.03 | .002 |  |  |  |  |  |  | 0.01 | -0.02 | 0.00 |  |
| Internalizing |  |  |  |  |  |  | -0.10 | 0.03 | .001 | -0.13 | -0.18 | -0.07 | -0.03 | -0.05 | -0.01 | 23% |
| Birth sex | 0.04 | 0.04 | .109 | 0.03 | 0.04 | .160 |  |  |  |  |  |  | 0.00 | 0.00 | 0.01 |  |
| Race/ethnicity | -0.16 | 0.04 | < .001 | -0.04 | 0.04 | .194 |  |  |  |  |  |  | 0.01 | -0.01 | 0.02 |  |
| Maternal education | 0.20 | 0.03 | < .001 | -0.14 | 0.04 | .001 |  |  |  |  |  |  | -0.03 | -0.04 | -0.01 |  |
| Socioeconomic index | 0.14 | 0.03 | < .001 | -0.06 | 0.04 | .034 |  |  |  |  |  |  | -0.01 | -0.02 | 0.00 |  |
| **Self-Reported Outcomes** |  |  |  |  |  |  |  |  |  |  |  |  |  |  |  |  |
| Social Competence |  |  |  |  |  |  | 0.05 | 0.03 | .062 | 0.10 | 0.03 | 0.16 | 0.05 | 0.03 | 0.07 | 50% |
| Birth sex | 0.04 | 0.04 | .129 | -0.02 | 0.04 | .353 |  |  |  |  |  |  | 0.00 | -0.01 | 0.00 |  |
| Race/ethnicity | -0.17 | 0.05 | < .001 | -0.10 | 0.05 | .021 |  |  |  |  |  |  | 0.02 | 0.00 | 0.04 |  |
| Maternal education | 0.20 | 0.03 | < .001 | 0.10 | 0.04 | .007 |  |  |  |  |  |  | 0.02 | 0.01 | 0.04 |  |
| Socioeconomic index | 0.14 | 0.03 | < .001 | 0.07 | 0.04 | .038 |  |  |  |  |  |  | 0.01 | 0.00 | 0.02 |  |
| Externalizing |  |  |  |  |  |  | -0.02 | 0.03 | .313 | -0.05 | -0.11 | 0.02 | -0.03 | -0.05 | -0.01 | 60% |
| Birth sex | 0.05 | 0.03 | .076 | 0.02 | 0.04 | .306 |  |  |  |  |  |  | 0.00 | 0.00 | 0.01 |  |
| Race/ethnicity | -0.16 | 0.04 | < .001 | 0.12 | 0.05 | .008 |  |  |  |  |  |  | -0.02 | -0.04 | 0.00 |  |
| Maternal education | 0.20 | 0.03 | < .001 | -0.02 | 0.04 | .321 |  |  |  |  |  |  | 0.00 | -0.02 | 0.01 |  |
| Socioeconomic index | 0.14 | 0.03 | < .001 | -0.07 | 0.04 | .041 |  |  |  |  |  |  | -0.01 | -0.03 | 0.00 |  |
| Internalizing |  |  |  |  |  |  | 0.00 | 0.03 | .495 | -0.01 | -0.08 | 0.05 | -0.01 | -0.03 | 0.01 | 100% |
| Birth sex | 0.05 | 0.04 | .075 | 0.05 | 0.04 | .113 |  |  |  |  |  |  | 0.00 | 0.00 | 0.01 |  |
| Race/ethnicity | -0.16 | 0.04 | < .001 | 0.03 | 0.05 | .259 |  |  |  |  |  |  | -0.01 | -0.02 | 0.01 |  |
| Maternal education | 0.20 | 0.03 | < .001 | -0.02 | 0.04 | .329 |  |  |  |  |  |  | 0.00 | -0.02 | 0.01 |  |
| Socioeconomic index | 0.14 | 0.03 | < .001 | -0.03 | 0.04 | .231 |  |  |  |  |  |  | 0.00 | -0.02 | 0.01 |  |
| **Objectively Measured Outcomes** | |  |  |  |  |  |  |  |  |  |  |  |  |  |  |  |
| Academic Skills |  |  |  |  |  |  | 0.13 | 0.03 | < .001 | 0.25 | 0.20 | 0.31 | 0.12 | 0.09 | 0.15 | 48% |
| Birth sex | 0.05 | 0.04 | .103 | 0.02 | 0.03 | .340 |  |  |  |  |  |  | 0.00 | 0.00 | 0.01 |  |
| Race/ethnicity | -0.16 | 0.04 | < .001 | -0.24 | 0.04 | < .001 |  |  |  |  |  |  | 0.04 | 0.02 | 0.06 |  |
| Maternal education | 0.20 | 0.03 | < .001 | 0.32 | 0.03 | < .001 |  |  |  |  |  |  | 0.06 | 0.04 | 0.08 |  |
| Socioeconomic index | 0.14 | 0.03 | < .001 | 0.12 | 0.03 | < .001 |  |  |  |  |  |  | 0.02 | 0.01 | 0.03 |  |

*Note. n* = 1,196. *B* = standardized beta coefficient estimate; *pSD* = posterior standard deviation; *p* = one-tailed *p*-value. Child sex was coded as 1 = male, 2 = female. Race/ethnicity was coded as 1 = White/non-Hispanic, 0 = non-White or Hispanic.

**Supplementary Table 22**

Path coefficients and indirect effects for mediation models with SSP 15 month as independent variable and demographic variables as mediator to adult academic achievement in the SECCYD

|  | Path A | | | Path B | | | Path C’ | | | Total Effect | | | Indirect Effects | | | I/T |
| --- | --- | --- | --- | --- | --- | --- | --- | --- | --- | --- | --- | --- | --- | --- | --- | --- |
|  |  |  |  |  |  |  |  |  |  |  | *95% Credible Interval* | |  | *95% Credible Interval* | |  |
|  | *B* | *pSD* | *p* | *B* | *pSD* | *p* | *B* | *pSD* | *p* | *B* | *Lower* | *Upper* | *B* | *Lower* | *Upper* |  |
| Academic Skills |  |  |  |  |  |  | 0.03 | 0.03 | .126 | 0.08 | 0.02 | 0.14 | 0.05 | 0.01 | 0.08 | 63% |
| Birth sex | 0.03 | 0.04 | .194 | 0.03 | 0.03 | .197 |  |  |  |  |  |  | 0.00 | 0.00 | 0.01 |  |
| Race/ethnicity | -0.07 | 0.04 | .065 | -0.27 | 0.04 | < .001 |  |  |  |  |  |  | 0.02 | 0.00 | 0.04 |  |
| Maternal education | 0.07 | 0.03 | .010 | 0.35 | 0.03 | < .001 |  |  |  |  |  |  | 0.02 | 0.00 | 0.04 |  |
| Socioeconomic index | 0.05 | 0.03 | .069 | 0.14 | 0.03 | < .001 |  |  |  |  |  |  | 0.01 | 0.00 | 0.02 |  |

*Note. n* = 1,191. *B* = standardized beta coefficient estimate; *pSD* = posterior standard deviation; *p* = one-tailed *p*-value. Child sex was coded as 1 = male, 2 = female. Race/ethnicity was coded as 1 = White/non-Hispanic, 0 = non-White or Hispanic. The adult academic achievement variable contains the childhood objective measures of academic achievement used in the main analyses as well as the *z*-standardized self-report of educational attainment in adulthood at 26 years of age.

**Supplementary Table 23**

Path coefficients and indirect effects for mediation models with 15-month Strange Situation Procedure with “cannot classify” cases disregarded as independent variable, demographic variables as mediators, and socioemotional and academic outcomes as dependent variables in the SECCYD

|  | Path A | | | Path B | | | Path C’ | | | Total Effects | | | Indirect Effects | | | I/T |
| --- | --- | --- | --- | --- | --- | --- | --- | --- | --- | --- | --- | --- | --- | --- | --- | --- |
|  |  |  |  |  |  |  |  |  |  |  | *95% Credible Interval* | |  | *95% Credible Interval* | |  |
|  | *B* | *pSD* | *p* | *B* | *pSD* | *p* | *B* | *pSD* | *p* | *B* | *Lower* | *Upper* | *B* | *Lower* | *Upper* |  |
| **Teacher-Reported Outcomes** | | | | | | | | | | | | | | | |  |
| Social Competence |  |  |  |  |  |  | 0.02 | 0.03 | .252 | 0.06 | 0.00 | 0.12 | 0.04 | 0.01 | 0.07 | 67% |
| Birth sex | 0.04 | 0.04 | .165 | -0.01 | 0.04 | .456 |  |  |  |  |  |  | 0.00 | -0.01 | 0.00 |  |
| Race/ethnicity | -0.08 | 0.04 | .026 | -0.24 | 0.04 | < .001 |  |  |  |  |  |  | 0.02 | 0.00 | 0.04 |  |
| Maternal education | 0.07 | 0.03 | .010 | 0.24 | 0.04 | < .001 |  |  |  |  |  |  | 0.02 | 0.00 | 0.03 |  |
| Socioeconomic index | 0.06 | 0.03 | .025 | 0.05 | 0.04 | .093 |  |  |  |  |  |  | 0.00 | 0.00 | 0.01 |  |
| Externalizing |  |  |  |  |  |  | -0.03 | 0.03 | .192 | -0.06 | -0.12 | 0.00 | -0.03 | -0.05 | -0.01 | 50% |
| Birth sex | 0.03 | 0.04 | .161 | -0.02 | 0.04 | .309 |  |  |  |  |  |  | 0.00 | -0.01 | 0.00 |  |
| Race/ethnicity | -0.08 | 0.04 | .020 | 0.24 | 0.04 | < .001 |  |  |  |  |  |  | -0.02 | -0.04 | 0.00 |  |
| Maternal education | 0.07 | 0.03 | .011 | -0.16 | 0.04 | < .001 |  |  |  |  |  |  | -0.01 | -0.02 | 0.00 |  |
| Socioeconomic index | 0.06 | 0.03 | .025 | -0.01 | 0.04 | .363 |  |  |  |  |  |  | 0.00 | -0.01 | 0.00 |  |
| Internalizing |  |  |  |  |  |  | -0.10 | 0.03 | .001 | -0.12 | -0.17 | -0.05 | -0.02 | -0.03 | 0.00 | 17% |
| Birth sex | 0.03 | 0.04 | .185 | 0.02 | 0.04 | .303 |  |  |  |  |  |  | 0.00 | 0.00 | 0.01 |  |
| Race/ethnicity | -0.08 | 0.04 | .023 | 0.06 | 0.05 | .123 |  |  |  |  |  |  | 0.00 | -0.02 | 0.00 |  |
| Maternal education | 0.07 | 0.03 | .010 | -0.15 | 0.04 | < .001 |  |  |  |  |  |  | -0.01 | -0.02 | 0.00 |  |
| Socioeconomic index | 0.06 | 0.03 | .023 | -0.05 | 0.04 | .095 |  |  |  |  |  |  | 0.00 | -0.01 | 0.00 |  |
| Academic Skills |  |  |  |  |  |  | 0.02 | 0.03 | .288 | 0.06 | -0.01 | 0.11 | 0.04 | 0.01 | 0.07 | 67% |
| Birth sex | 0.04 | 0.04 | .165 | -0.05 | 0.04 | .062 |  |  |  |  |  |  | 0.00 | -0.01 | 0.00 |  |
| Race/ethnicity | -0.09 | 0.04 | .014 | -0.20 | 0.04 | < .001 |  |  |  |  |  |  | 0.02 | 0.00 | 0.04 |  |
| Maternal education | 0.07 | 0.03 | .010 | 0.28 | 0.03 | < .001 |  |  |  |  |  |  | 0.02 | 0.00 | 0.04 |  |
| Socioeconomic index | 0.06 | 0.03 | .022 | 0.09 | 0.04 | .004 |  |  |  |  |  |  | 0.01 | 0.00 | 0.01 |  |
| **Mother-Reported Outcomes** |  |  |  |  |  |  |  |  |  |  |  |  |  |  |  |  |
| Social Competence |  |  |  |  |  |  | 0.06 | 0.03 | .030 | 0.08 | 0.03 | 0.15 | 0.03 | 0.01 | 0.06 | 38% |
| Birth sex | 0.04 | 0.04 | .180 | -0.08 | 0.04 | .014 |  |  |  |  |  |  | 0.00 | -0.01 | 0.00 |  |
| Race/ethnicity | -0.08 | 0.04 | .016 | -0.22 | 0.04 | < .001 |  |  |  |  |  |  | 0.02 | 0.00 | 0.04 |  |
| Maternal education | 0.07 | 0.03 | .009 | 0.19 | 0.04 | < .001 |  |  |  |  |  |  | 0.01 | 0.00 | 0.03 |  |
| Socioeconomic index | 0.06 | 0.03 | .020 | 0.05 | 0.04 | .103 |  |  |  |  |  |  | 0.00 | 0.00 | 0.01 |  |
| Externalizing |  |  |  |  |  |  | 0.01 | 0.03 | .405 | -0.01 | -0.07 | 0.05 | -0.02 | -0.04 | 0.00 | N/A* |
| Birth sex | 0.04 | 0.04 | .160 | 0.04 | 0.04 | .162 |  |  |  |  |  |  | 0.00 | 0.00 | 0.01 |  |
| Race/ethnicity | -0.09 | 0.04 | .016 | -0.02 | 0.04 | .308 |  |  |  |  |  |  | 0.00 | -0.01 | 0.01 |  |
| Maternal education | 0.07 | 0.03 | .012 | -0.23 | 0.04 | < .001 |  |  |  |  |  |  | -0.02 | -0.03 | 0.00 |  |
| Socioeconomic index | 0.06 | 0.03 | .023 | -0.08 | 0.04 | .012 |  |  |  |  |  |  | 0.00 | -0.01 | 0.00 |  |
| Internalizing |  |  |  |  |  |  | -0.01 | 0.03 | .351 | -0.02 | -0.08 | 0.04 | -0.01 | -0.03 | 0.00 | 50% |
| Birth sex | 0.04 | 0.04 | .154 | 0.02 | 0.04 | .313 |  |  |  |  |  |  | 0.00 | 0.00 | 0.01 |  |
| Race/ethnicity | -0.09 | 0.04 | .013 | -0.03 | 0.04 | .277 |  |  |  |  |  |  | 0.00 | -0.01 | 0.01 |  |
| Maternal education | 0.07 | 0.03 | .012 | -0.15 | 0.04 | < .001 |  |  |  |  |  |  | -0.01 | -0.02 | 0.00 |  |
| Socioeconomic index | 0.06 | 0.03 | .024 | -0.05 | 0.04 | .063 |  |  |  |  |  |  | 0.00 | -0.01 | 0.00 |  |
|  |  |  |  |  |  |  |  |  |  |  |  |  |  |  |  |  |
| **Self-Reported Outcomes** |  |  |  |  |  |  |  |  |  |  |  |  |  |  |  |  |
| Social Competence |  |  |  |  |  |  | 0.06 | 0.03 | .032 | 0.08 | 0.01 | 0.15 | 0.02 | 0.00 | 0.04 | 25% |
| Birth sex | 0.03 | 0.04 | .198 | -0.01 | 0.04 | .373 |  |  |  |  |  |  | 0.00 | -0.01 | 0.00 |  |
| Race/ethnicity | -0.08 | 0.04 | .040 | -0.11 | 0.05 | .018 |  |  |  |  |  |  | 0.01 | 0.00 | 0.02 |  |
| Maternal education | 0.07 | 0.03 | .008 | 0.09 | 0.04 | .013 |  |  |  |  |  |  | 0.01 | 0.00 | 0.02 |  |
| Socioeconomic index | 0.06 | 0.03 | .027 | 0.08 | 0.04 | .031 |  |  |  |  |  |  | 0.00 | 0.00 | 0.01 |  |
| Externalizing |  |  |  |  |  |  | -0.07 | 0.03 | .026 | -0.08 | -0.15 | -0.01 | -0.01 | -0.03 | 0.00 | 13% |
| Birth sex | 0.04 | 0.04 | .148 | 0.05 | 0.04 | .141 |  |  |  |  |  |  | 0.00 | 0.00 | 0.01 |  |
| Race/ethnicity | -0.08 | 0.04 | .021 | 0.12 | 0.05 | .011 |  |  |  |  |  |  | -0.01 | -0.03 | 0.00 |  |
| Maternal education | 0.07 | 0.03 | .007 | -0.03 | 0.04 | .252 |  |  |  |  |  |  | 0.00 | -0.01 | 0.00 |  |
| Socioeconomic index | 0.06 | 0.03 | .019 | -0.07 | 0.04 | .051 |  |  |  |  |  |  | 0.00 | -0.01 | 0.00 |  |
| Internalizing |  |  |  |  |  |  | -0.04 | 0.03 | .117 | -0.04 | -0.11 | 0.03 | 0.00 | -0.01 | 0.01 | 0% |
| Birth sex | 0.04 | 0.04 | .156 | 0.08 | 0.04 | .036 |  |  |  |  |  |  | 0.00 | 0.00 | 0.01 |  |
| Race/ethnicity | -0.08 | 0.04 | .024 | 0.02 | 0.05 | .393 |  |  |  |  |  |  | 0.00 | -0.01 | 0.01 |  |
| Maternal education | 0.07 | 0.03 | .009 | -0.01 | 0.04 | .428 |  |  |  |  |  |  | 0.00 | -0.08 | 0.01 |  |
| Socioeconomic index | 0.06 | 0.03 | .017 | -0.04 | 0.04 | .199 |  |  |  |  |  |  | 0.00 | -0.01 | 0.00 |  |
| **Objectively Measured Outcomes** | | | | | | | | | | | | | | | | |
| Academic Skills |  |  |  |  |  |  | 0.02 | 0.03 | .190 | 0.08 | 0.02 | 0.13 | 0.05 | 0.02 | 0.09 | 63% |
| Birth sex | 0.04 | 0.04 | .142 | 0.02 | 0.03 | .308 |  |  |  |  |  |  | 0.00 | 0.00 | 0.01 |  |
| Race/ethnicity | -0.08 | 0.04 | .013 | -0.26 | 0.04 | < .001 |  |  |  |  |  |  | 0.02 | 0.00 | 0.04 |  |
| Maternal education | 0.07 | 0.03 | .007 | 0.33 | 0.03 | < .001 |  |  |  |  |  |  | 0.02 | 0.00 | 0.04 |  |
| Socioeconomic index | 0.06 | 0.03 | .019 | 0.13 | 0.03 | < .001 |  |  |  |  |  |  | 0.01 | 0.00 | 0.02 |  |

*Note. n* = 1,149. *B* = standardized beta coefficient estimate; *pSD* = posterior standard deviation; *p* = one-tailed *p*-value. Child sex was coded as 1 = male, 2 = female. Race/ethnicity was coded as 1 = White/non-Hispanic, 0 = non-White or Hispanic. *As described by Wen & Fan (2015), in mediation models where the direct effect (C') and the indirect effect have opposite signs, the I/T statistic is not calculable or interpretable because the indirect effect could be any value and has no theoretical bounds.
